# High-Throughput Hyperspectral and Multiplexed Super-Resolution Fluorescence Imaging by SP-STORM

**DOI:** 10.1021/jacsau.5c01677

**Published:** 2026-01-21

**Authors:** Elric Dion Pott, Meek Yang, James Ethan Batey, Joie Embree, Bin Dong

**Affiliations:** Department of Chemistry and Biochemistry, 3341 University of Arkansas, Fayetteville, Arkansas 72701, United States

**Keywords:** High throughput, Single molecule spectroscopy, Spectral phasor, Hyperspectral super-resolution imaging, 5-plexed 3D SMLM in parallel

## Abstract

Simultaneous determination of spatial location and spectral color of single molecules at large molecular density with high throughput was achieved by combining single-molecule photoswitching and optical in-hardware Fourier transformation of single-molecule emission spectra into the phasor space. The method, named as spectral phasor enabled stochastic optical reconstruction microscopy (SP-STORM), achieved simultaneous super-resolution imaging of five subcellular structures in parallel with minimum crosstalk for the first time. The high-throughput feature of SP-STORM enables these subcellular structures to be readily resolved in about one min, which is more than an order of magnitude faster than other multiplexing single-molecule localization microscopy techniques. The concept of SP-STORM is also compatible with and can be readily applicable to other super-resolution microscopy.

## Graphical abstract

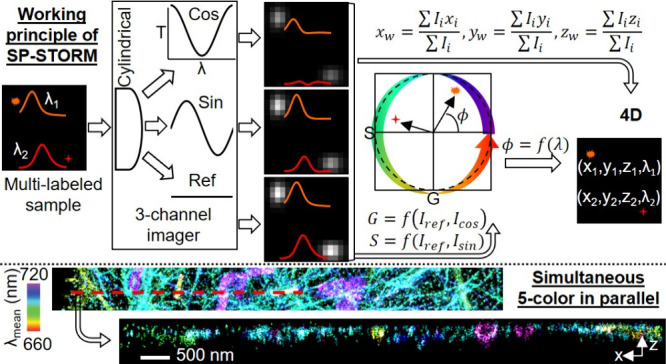

Single-molecule localization-based super-resolved fluorescence microscopy (SMLM)^1,2^ has advanced studies of protein assemblies down to nanometer spatial resolution and even at single-protein resolution.^3,4^ Further advancements in multicolor SMLM attempt to explore its multiplexing capability. Utilizing spectrally well-separated fluorophores^5,6^ is the most straightforward approach, yet it requires long data acquisition time due to sequential image registration and involves complex error-prone alignment procedures between channels. A ratiometric-based spectral demixing approach enables simultaneously resolving up to four targets but suffers high crosstalk.^7^ Dispersive (i.e., SR-STORM^8,9^ ) and excitation modulation (i.e., ExR-STORM^10^) based spectral demixing approaches can simultaneously resolve four targets with low crosstalk. However, both suffer low throughput due to either a low molecular density requirement to avoid spectral interference (SR-STORM) or mechanical switching between excitation laser lines (ExR-STORM). Four-color SR-STORM and ExR-STORM take 12 and 25 min, respectively. In comparison, single-color dSTORM requires less than a minute.^11,12^

DNA-PAINT is another type of multiplexed SMLM with high spatial resolution.^13^ It has theoretically unlimited multiplexing capability in combination with solution exchange (Exchange-PAINT).^14^ However, the throughput of this approach remains a major limitation. Recent optimizations, such as FRET-based probes^15^ and fluorogenic DNA-PAINT,^16^ have improved acquisition speed at the expense of reducing multiplexing capacity. The development of secondary label-based unlimited multiplexed PAINT (SUM-PAINT)^17^ attempted to address this trade-off by decoupling the DNA barcoding of the target from the imaging process. The time to quantitatively map up to 30 proteins in neuron cells at single-protein resolution was reduced from 800 h using Exchange-PAINT^14^ to 30 h using SUM-PAINT. Nevertheless, the intrinsic nature of sequential imaging in SUM-PAINT remains as the bottleneck for high-throughput analysis.

Here, we developed a nondispersive approach to simultaneously determine single-molecule locations and their fluorescence emission spectra with high throughput. The approach is based on the integration of single-molecule imaging and optical in-hardware Fourier transformation of single molecules’ emission spectra into the phasor space for spectral demixing (i.e., in-hardware spectral phasor analysis). In this work, we integrate the in-hardware spectral phasor analysis with dSTORM^11^ imaging, named as SP-STORM. We simultaneously obtained multiplexed super-resolution microscopy image of 5 protein targets in parallel and with minimum crosstalk in ∼1 min. This is more than an order of magnitude faster than all previously developed multiplexed SMLM techniques which can simultaneously resolve up to 4 protein targets in few tens of minutes to hours. We further demonstrated SP-STORM’s compatibility for 3D super-resolution imaging.

In SP-STORM, we modulated single molecules’ fluorescence emission using a lab-built three-channel imager (Figure 1a, Figure S1), where the signals in two of the channels were transformed by cosine- and sine-shaped optical bandpass filters (Figure 1a, inset), namely, the sine and cosine channels. The unmodified signal in the remaining channel works as the reference for phasor analysis. Using the photon number of the same single molecules in three channels, one can obtain their locations in the phasor space (Figure 1, S2) where the phase angle (ϕ) is wavelength-dependent. We calibrated the imaging system to establish the spectral mean-phase angle relationship by refining the spectral color of light from a microscope built-in lamp or emission from fluorescence beads using narrow bandpass filters (Figure 1b-d). The obtained spectral mean-phase angle relationship follows a nonlinear trend that was well-fit by a fourth-order polynomial function (Figure 1e). This calibration curve was then used to determine the spectral mean of single molecules in dSTORM imaging experiments. Notably, optical filters with transmission profiles representing complete sine and cosine functions are ideal.^18,19^ However, optical filters with broadband transmission similar to ideal sine/cosine filters can achieve the same purpose with a modified analysis procedure (Figure S3).^20^ Nevertheless, we speculated that using nonideal transmission optical filters could result in larger errors in the obtained spectral information. Using simulation data, we evaluated such impact, and the results suggest that the error from using nonideal optical filters is minimum (Additional discussion in Figure S4).

**Figure 1:**
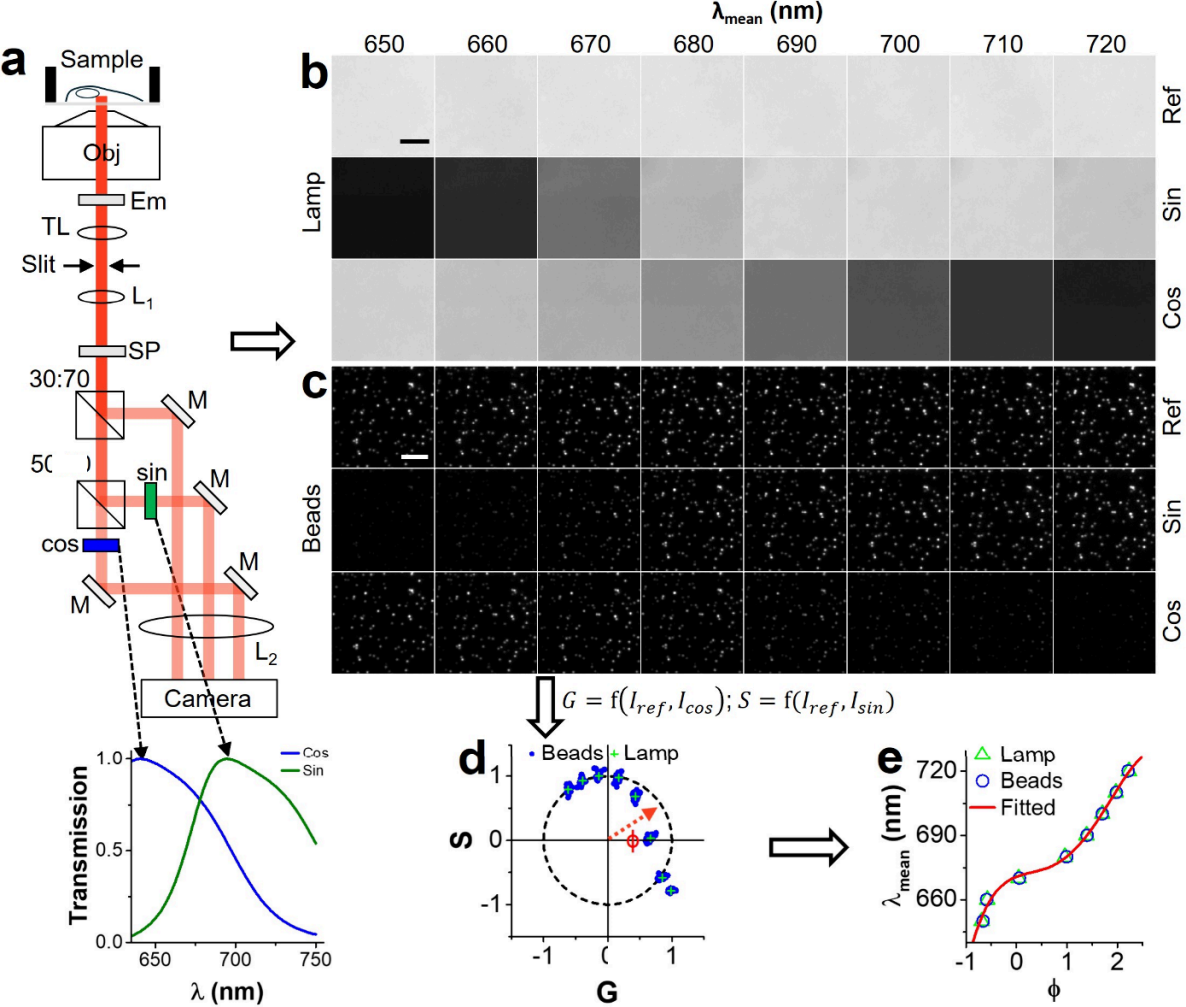
**Working principle of SP-STORM.** (a) Schematic of the optical setup for emission detection. DC, dichroic mirror; Em, long pass emission filter; TL, tube lens; L, lens; SP, short pass filter; and M, mirror. Inset: Normalized transmission profiles of adapted sine and cosine filters in the confined wavelength range. (b) Brightfield image of microscope lamp light and (c) fluorescence image of beads at different wavelengths. (d) Phasor plot of results at different wavelengths. (e) The wavelength-phase angle relationship for system calibration. The data was fitted by a fourth-order polynomial function. Scale bar: 5 μm.

We first tested the widely used dSTORM dye Alexa fluor 647 (AF647) for SP-STORM imaging. We labeled the fixed cells through immunostaining and then photoswitched most of AF647 dye molecules into a nonfluorescent dark state (i.e., off state) resulting in only a subset of dye molecules fluorescing (i.e., on state) at any given instant. By optimizing the excitation laser power density (>10 kW cm^–2^) and imaging acquisition rate (200 Hz), the transformed (i.e., cosine and sine) and unmodified (i.e., reference) emission signals from sparsely distributed single AF647 molecules were simultaneously recorded at different regions of the same camera (Figure 2a). The same single AF647 molecules in three channels were localized and grouped. Their photon numbers were measured and used to determine single AF647 molecules’ phase angles in the phasor space (Figure 2b), which enables the quantification of single-molecule spectral means using the established calibration curve (Figure 1e). The targeted subcellular structures were then reconstructed using the weighted spatial locations of single AF647 molecules in three channels with colors representing the single-molecule spectral mean (Figure 2c). The localization precision for AF647 was estimated to be ∼ 6 nm from small cluster analysis (Figure S7), which corresponds to a spatial resolution of ∼ 15 nm (full width at half-maximum) comparable to typical dSTORM imaging. The histogram distribution of >10^5^ single AF647 molecules (Figure 2d) gives an average spectral mean of 674.2 nm and a spectral variation of 1.4 nm (s.d.). Such small spectral variation holds promise for highly multiplexed SMLM by spectral demixing dyes with minimal spectral differences.

**Figure 2:**
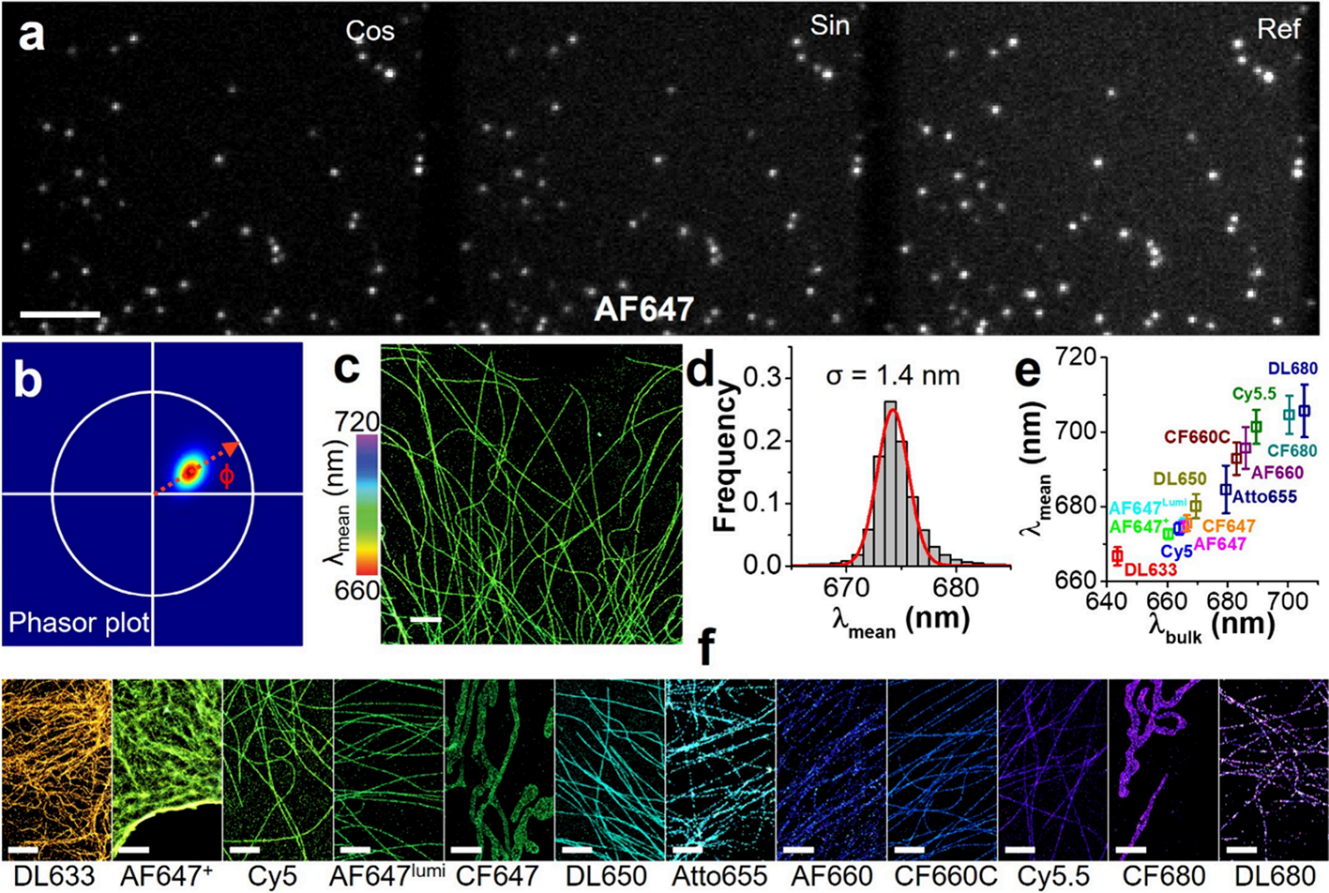
**SP-STORM imaging with 13 far-red dyes.** (a) Simultaneous acquiring of transformed (i.e., sine/cosine) and unmodified (i.e., ref) images from the same single AF647 molecules. (b) Phasor plot of >10^5^ single AF647 molecules. (c) Super-resolution image of labeled microtubule with color representing single-molecule spectral mean. (d) 1D Gaussian fitting the histogram of the spectral mean of single AF647 molecules gives an average of 674.2 ± 1.4 nm (mean ± s.d.). (e) Measured spectral mean for single molecules of 13 tested far-red dyes. Error bars are s.d. from Gaussian fitting of the histogram distribution of spectral mean from single molecules (>10^5^ for each dye). (f) Super-resolution image of labeled subcellular structures for different dyes, with color representing their single-molecule spectral mean. Scale bars: (a) 5 μm and (c, f) 2 μm.

However, significant broadening and fluctuation of spectral color at single molecule level has been well-known due to factors including photon noise, background noise, sensitivity of the detector, and inherent heterogeneity. It has been reported that single molecules can show a spectral shift of up to 30 nm in emission peak.^9,21^ We evaluated another 12 far-red organic dyes (λ_bulk_: 644–706 nm, Figure S5–S17). Representative SP-STORM images are shown in Figure 2f. The spatial resolution ranges from 15 to 40 nm. The results of the single-molecule spectral mean and variation for each dye are shown in Figure 2e. Dye molecules with bulk emission peak differences as small as 5 nm (e.g., AF647 vs DL650) can be readily resolved by SP-STORM due to their small single-molecule spectral variations. Therefore, theoretically, approximately 23 dyes can be spectrally resolved by SP-STORM in the spectral window of 635–750 nm, assuming all dyes own such small single-molecule spectral variation and can be efficiently excited and photoswitched by a single 628 nm laser. However, the results show a spectral variation ranging from 1.3 to 7.1 nm for all tested far-red dyes, with a general trend of smaller single-molecule spectral variation for brighter dyes (Figure S18). Furthermore, most of the tested dyes show a single population of spectral color, except AF660 and DL680 (Figures S13, S17), highlighting the importance of assessing the inherent heterogeneity of dye molecules at the single molecule level. Importantly, these screening results laid the foundation for multiplexed SMLM by choosing dyes based on photoswitching capability, spatial resolution, spectral variation, and overlapping in the phasor space.

To test SP-STORM for multiplexed imaging and compare its throughput to other methods, we labeled four distinct subcellular targets in fixed cells with four dyes of heavily overlapped emission spectra. A single 628 nm laser was used for exciting and photoswitching all dye molecules between on and off states (Figure 3a). Excitingly, the targeted four subcellular structures are readily distinguishable based on the spectral mean alone (Figure 3c). Distinct colors of purple, blue, green, and yellow were clearly observed for labeled mitochondria, microtubule, vimentin, and peroxisome, respectively. Single molecules were further classified for multiplexed images by comparing their locations in the phasor space to that of known dye molecules (Figure S19g). dSTORM images of labeled subcellular structures show negligible misidentification (Figure 3e-i, Figure S19b-f) with color crosstalk <2% (Figure 3j). We then evaluated the throughput of SP-STORM. The number of locations per image frame along with the data acquisition time was plotted in Figure 3b. The average number of localizations per image frame was estimated to be ∼ 74, corresponding a density of ∼ 0.14 locations per μm^2^. Under this density of single molecules, multiplexed dSTORM images with highly resolvable subcellular structures are already readily available in less than a minute (Figure 3d) based on the Nyquist limit^22^ (Figure S20).

**Figure 3:**
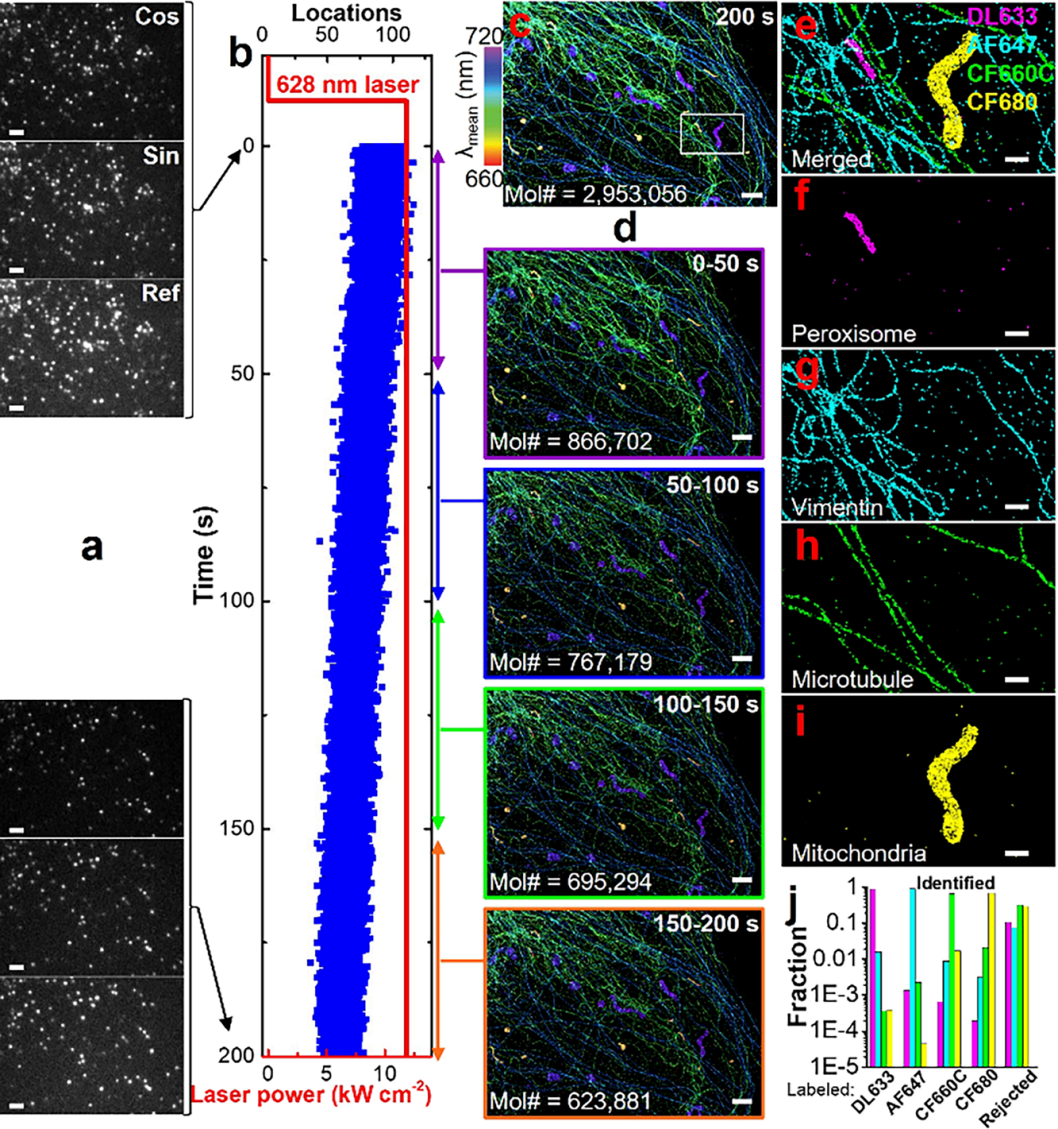
**SP-STORM enables hyperspectral and simultaneously multiplexed imaging with high throughput.** (a) First and last image frames of single molecules in four-color SP-STORM. (b) Trajectory of number of locations and laser power density versus time. (c) Hyperspectral dSTORM image of four proteins (i.e., PMP70, vimentin, α-tubulin, TOM20) labeled by DL633, AF647, CF660C, and CF680 in a fixed COS-7 cell. The color denotes the spectral mean of single molecules. (d) Hyperspectral dSTORM image for time segments of 50 s achieving minimal density based on the Nyquist criterion. Mol# represents total number of molecular locations obtained for image reconstruction. (e-i) dSTORM images of four proteins after classification of single molecules. (j) Color crosstalk between channels. The rejected fraction represents data outside the defined boundary conditions in the phasor space (Figure S19g). Scale bars: 2 μm (a,c,d) and 500 nm (e-i).

We further tested the feasibility of SP-STORM for simultaneous super-resolution imaging of five targets (Figure 4). Five subcellular structures are readily observed with distinct colors (Figure 4a), where purple, blue, cyan, yellow-green, and yellow denote mitochondria, microtubule, vimentin, actin, and peroxisome, correspondingly. The multiplexed dSTORM image was readily obtained in ∼ 1.3 min (Figure S21). For all channels, the crosstalk was determined to be <5% (Figure 4b-i). Simultaneous 5-color SMLM imaging in parallel has never been achieved previously. To evaluate if the superlocalized positions align well for different dye molecules, we performed SP-STORM imaging of mitochondrial outer membrane protein (i.e., TOM20) and mitochondrial heat shock protein (i.e., mtHsp70 or Mortalin, primarily localizes inside the mitochondria) with CF680 and DL650. The hyperspectral and multiplexed dSTORM images (Figure 4j,k) and the cross-section profile (Figure 4l) clearly match the expectation that locations of DL650 are fully surrounded by CF680.

**Figure 4:**
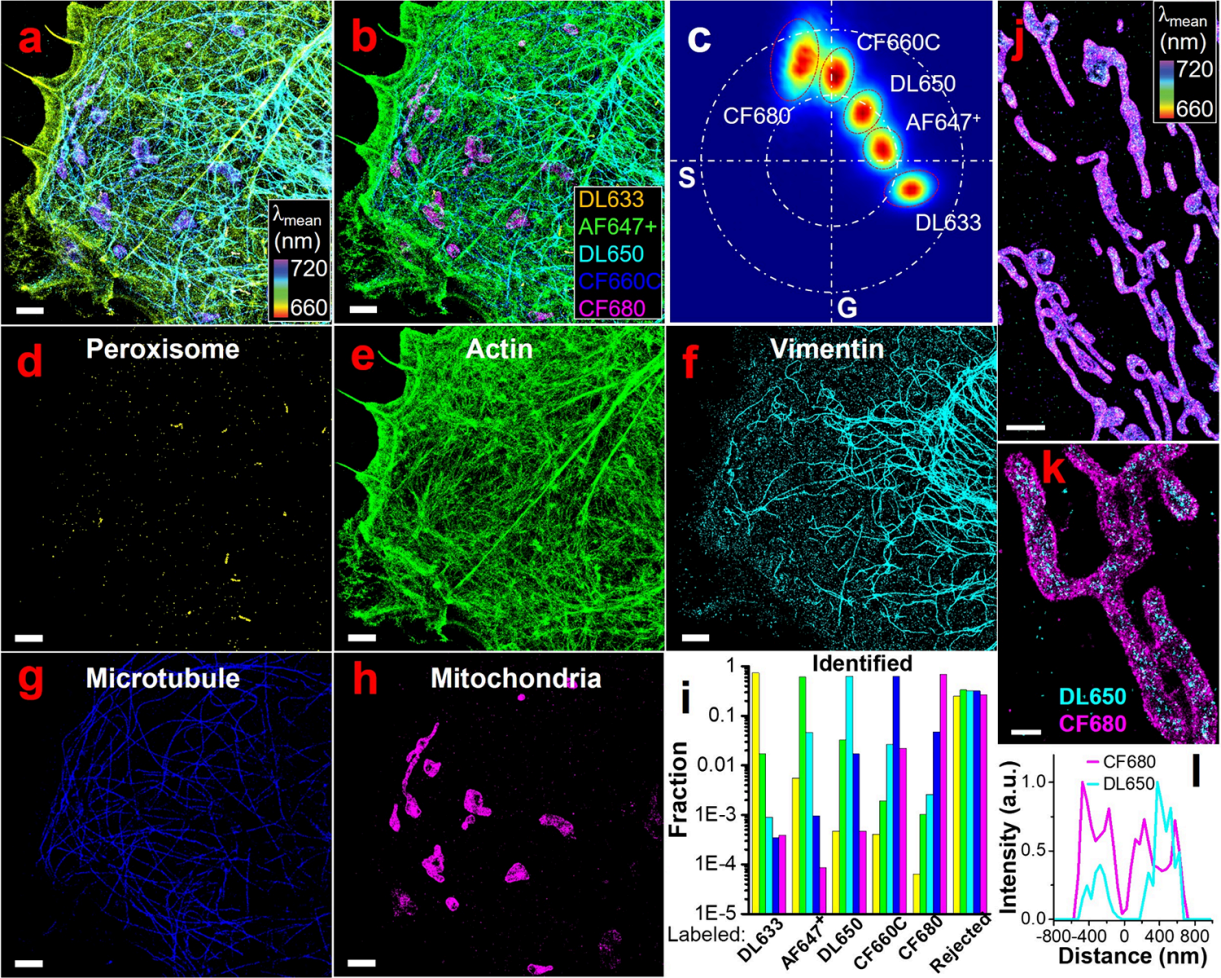
**5-color SP-STORM.** (a) Hyperspectral dSTORM image of five proteins (i.e., PMP70, F-actin, vimentin, α-tubulin, TOM20) labeled by DL633, AF647^+^, DL650, CF660C, and CF680 in a fixed COS-7 cell. (b) 5-color dSTORM image with single molecules being classified and recolored. The classification was based on the comparison of single molecules’ location in phasor space to five dyes. (c) Boundary conditions in phasor plot were established from single-color labeled sample and used to separate the different dye molecules. The red dashed dotted line denotes 1.2–2.0 s.d. of distributions for each dye. The white dash dot line is for illustration location of dyes in the phasor plot with large and small circles as unit and half-unit circle, respectively. (d-h) The separated channels of five subcellular structures. (i) Color crosstalk between channels. (j) Hyperspectral and (k) 2-color dSTORM images (white box in (j)) of TOM20 and mortalin labeled with CF680 and DL650, respectively. (l) Cross-section profile of two dyes along the long axis of white box in (k). Scale bars: 2 μm (a-h, j) and 500 nm (k).

We next demonstrated the capability of SP-STORM for 3D super-resolution imaging. Here, we adapted astigmatism-based 3D SMLM technique^23^ by introducing a weak cylindrical lens (1000 mm focal length) at approximately 2 cm away from the intermediate image plane (Figures S1, S22). By combining the spectral and spatial information, a 4D (i.e., x, y, z, and λ) super-resolution image was obtained (Figure 5). The low color crosstalk remains, where negligible misidentification between five dye channels was observed (Figure 5b-f). Notably, here 3D dSTORM images were presented in individual dye channels. The 3D localization precision of sub-10 nm (lateral) and sub-20 nm (axial) were achieved for all dyes (Figure S23), similar to previously reported results.^23^ Figure 5g shows a cross-section image in a xz plane, providing a more complete visualization of spatial arrangement and interaction of targeted subcellular structures. We selected three regions for cross-section profile analysis of the distributions of five super-resolved structures as examples: the nanoscale layer-by-layer distribution of cytoskeleton filaments at site #1, the contact of microtubules on the top of a mitochondrion at site #2, and the insertion of peroxisome between layers of cytoskeleton filaments at site #3.

**Figure 5:**
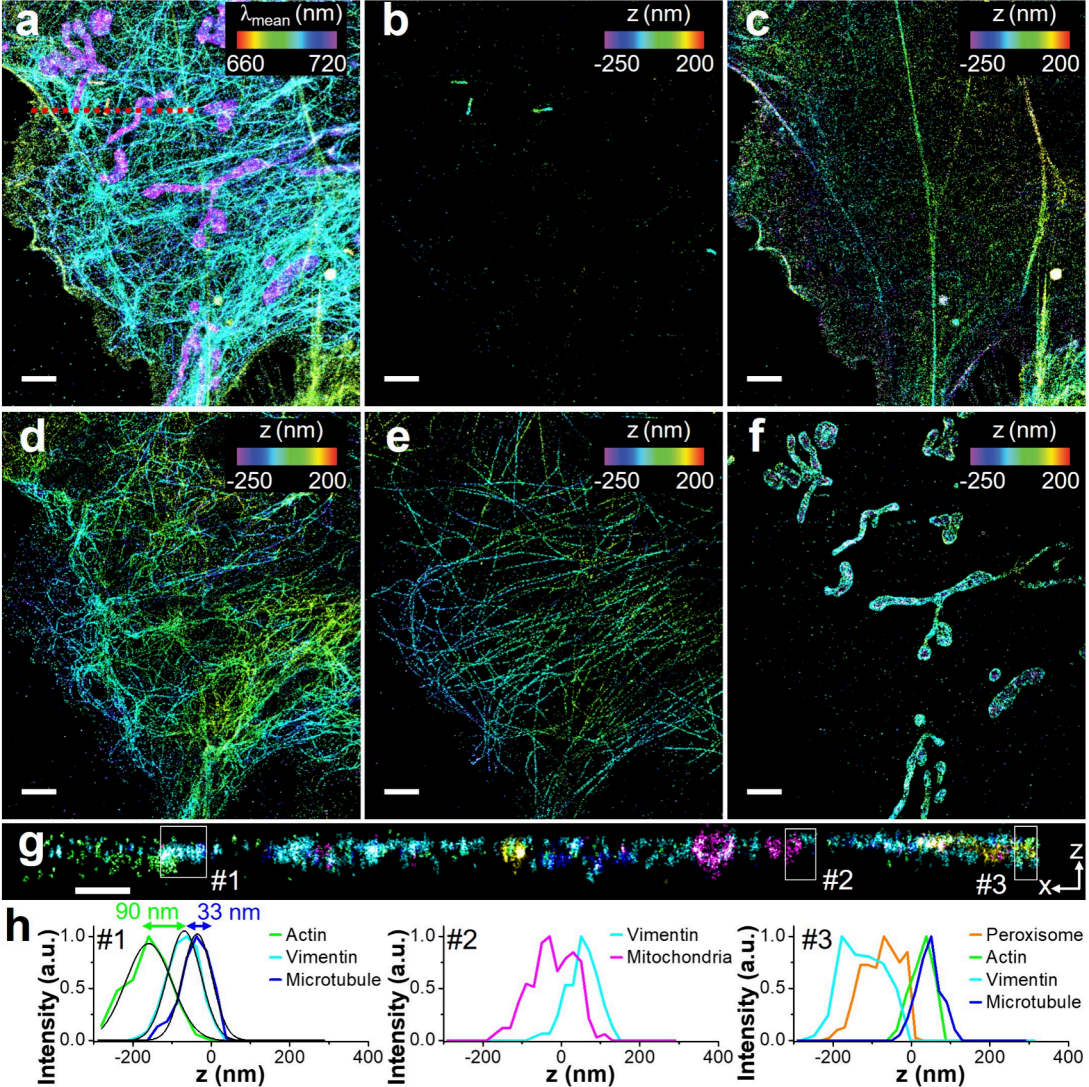
**5-color 3D SP-STORM.** (a) Hyperspectral dSTORM image of five proteins (i.e., PMP70, F-actin, vimentin, α-tubulin, TOM20) labeled by DL633, AF647^+^, DL650, CF660C, and CF680 in a fixed COS-7 cell. (b-f) 3D dSTORM images of separated channels of five subcellular structures. (g) A cross section in xz plane along the red dash line highlighted in (a). (h) Cross-section profile of dyes along the *z* axis of three white boxes highlighted in (g). Fitting the distribution of different cytoskeleton filaments with a 1D Gaussian function (black solid line) at site #1 showcases the resolved nanoscale gaps between filament types. Scale bars: 2 μm (a-f) and 500 nm (g).

The key challenges in advancing techniques for quantifying biomolecules and their spatial locations include sensitivity, spatial resolution, multiplexing capability, and throughput. Previously, the advancement of SMLM techniques enabled the simultaneous measurement of up to four protein targets in parallel or sequential quantification of theoretically unlimited protein targets with nanometer spatial resolution. However, the intrinsic nature of these techniques, either low SNR, low molecular density, low data acquisition rate, or sequential data acquisition, limits their throughput. The unique strength of SP-STORM lies in its advantages of a nondispersive approach and using cosine- and sine-shaped broadband optical filters. It enables high-density single-molecule imaging with high SNR resulting in a low spectral uncertainty of measurement, which ultimately leads to the high throughput and large multiplexing capability of SP-STORM. SP-STORM has demonstrated the capacity to simultaneously resolve five distinct protein targets in parallel and with low crosstalk in about a minute, which is more than an order of magnitude faster than all current multiplexed SMLM techniques (i.e., a few tens of minutes to hours for four protein targets). The advantages and limitations of current multiplexed SMLM techniques including SP-STORM were summarized in Table S1.

While it is beyond the scope of the current work, SP-STORM is readily adaptable for other applications, such as studying the transformation of intermediates or isomers in chemical reactions where distinct emission profiles are present, and sensing local environment at nanoscale using environmental sensitive dyes (e.g., solvatochromic dye, Nile red). Furthermore, the high-throughput feature of SP-STORM also holds promises for studying dynamic processes in live cells. By applying high laser power (>10 kW cm^–2^) and fast data acquisition speed (∼kHz), single-color dSTORM imaging of live cell membrane dynamics was achieved with 1–2 s temporal resolution.^12^ However, one needs to carefully assess the potential phototoxicity when operating under such a high illumination intensity. Additionally, motion blur due to sample drift can potentially introduce artifacts into the results of cellular dynamic processes. One can add nonphotobleaching fiducial markers (e.g., gold nanoparticles) to either lock the sample in three-dimensional or correct drift in post data analysis. Lastly, the integration of spectral phasor analysis for spectral demixing of fluorophores is broadly compatible with other super-resolution techniques, e.g., (F) PALM, PAINT, and STED, highlighting its versatility and potential for advancing multiplexed microscopy across various imaging platforms. However, it is worth noting that applying spectral phasor analysis for microscopy imaging with bulk emission^24^ (e.g., STED) potentially risks high color crosstalk for dyes with heavily overlapped spectra.

## Supplementary Material



## Data Availability

All data needed to evaluate the conclusions in the paper are presented in the paper and/or Supporting Information. Additional data related to this paper are available from the corresponding author on reasonable request.

## Notes

Experimental methods, materials, additional results and discussion, and supporting figures (PDF)
