# Supplementary material for: High-Throughput Hyperspectral and Multiplexed Super-Resolution Fluorescence Imaging by SP-STORM
Source: JACS Au. 2026 Jan 21;6(2):788–94. doi: 10.1021/jacsau.5c01677 (PMC12933340; doi:10.1021/jacsau.5c01677)
Supplement: Supplementary file 1 [file au5c01677_si_001.pdf]

Supporting Information

High Throughput Hyperspectral and Multiplexed Super-Resolution Fluorescence Imaging by SP-STORM

Elric Dion Pott[§], Meek Yang[§], James Ethan Batey, Joie Embree, Bin Dong*

Department of Chemistry and Biochemistry, University of Arkansas, Fayetteville, Arkansas 72701, United States.

[§]These authors contributed equally to this work.

*To whom correspondence should be addressed. E-Mail: bind@uark.edu.

Table of Contents

S1. The Optical setup. 2

S2. Spectral calibration. 2

S3. Dyes and antibodies. 3

S4. Sample preparation. 3

S4.1. Cell culture. 3

S4.2. Cell labelling with far-red dyes. 3

S5. SP-STORM imaging. 4

S6. Data analysis. 4

S7. Simulation study. 5

S7.1. Using ideal sine/cosine transmission filters. 6

S7.2. Actual sin/cos filters. 7

S8. Supporting Figures. 8

S9. Supporting Tables. 31

S10. References 32

S1. The Optical setup.

The SP-STORM was carried out on an Olympus IX-81 inverted microscope (Figure S1) equipped with an oil immersion objective (UPLAPO100X, NA 1.50, Olympus). 405 nm (Coherent) laser was coupled into an optical fiber, recollimated by a fiber coupler, and focused on the back focal plane of the oil immersion objective by a lens (L5) after passing a pair of relay lens (L3, L4). Laser at 628 nm (MPB Communications) was combined into the same optical path of 405 nm laser line using a short pass dichroic mirror (DC1) after passing a pair of relay lens (L1, L2). Lasers were directed into the sample by a dichroic mirror (DC2). All Lasers were cleaned by narrow bandpass filters (Ex1 and Ex2). Two translation stages were used for shifting the laser beams laterally before entering the objective so that the laser beams project to the sample/coverslip interface at an angle slightly smaller than the critical angle. The emission signals from dye molecules were collected by the same oil immersion objective. A long pass emission filter (RET638lp, Chroma) was used to reject laser scattering background. Then, the collected signal was directed to a lab-built three-channel imager for in-hardware transformation based spectral phasor analysis. Inside the three-channel imager, the emission signal from dye molecules was first split by a 30:70 (R:T) nonpolarized beam splitter (BS019, Thorlabs). The transmitted light after the first beam splitter was split further by a 50:50 (R:T) nonpolarized beam splitter (BS013, Thorlabs). The reflected and transmitted signals from the second beam splitter were transformed by sine and cosine function-like optical filters respectively (See details in Figure S1). Three images of the same dye molecules, i.e., reference, sin/cos-modified images, were projected on different regions of the same electron-multiplying charge-coupled device (EMCCD) camera (iXon Ultra 897, Andor) using a pair of relay lens (L6, L7). A short pass optical filter (FESH0750, Thorlabs) was used to confined spectral window below 750 nm. An optical slit was placed at the intermediate image plane of tube lens allowing crop field of view and avoiding crosstalk between three channels. The sample stage was locked by a self-built autofocusing optical system running by self-written Python scripts.

S2. Spectral calibration.

We applied two approaches to calibrate the wavelength-phase angle relationships of the SP-STORM imaging system. In one approach, brightfield images of transmitted light from the microscope lamp were captured. The lamp light was filtered by a series of narrow bandpass filters (Thorlabs) giving three images (i.e., reference, sine-, and cosine-modified) at specific wavelengths (Figure 1b). Measuring the intensities from three channels allows the determination of locations of spectral color in the phasor plot (Figure 1d). In another approach, 40 nm dark red fluorescent beads (F8789, ThermoFisher) were drop-casted on a coverslip at low density. The sample loaded with fluorescent beads was mounted on the microscope and excited by the lasers at a weak intensity. Similarly, narrow bandpass filters (Thorlabs) were inserted in the detection optical pathways. Three images (i.e., reference, sine-, and cosine-modified) of the fluorescent emission from the same single beads at specific wavelengths were captured (Figure 1c). The

locations and photon number of single fluorescent beads in all three channels were super-localized and determined. The same fluorescent beads in three channels were identified through previous developed image projection procedure.[1] The location of specific wavelength in the phasor plot was calculated by the photon number of beads in three channels (Figure 1d). Results from both methods generated similar calibration curves that were well fitted by a fourth-order polynomial function (Figure 1e).

S3. Dyes and antibodies.

These dyes were obtained from ThermoFisher (DyLight633, DyLight650, DyLight680, Alexa Fluor 647, and Alexa Fluor 660) as gifts and purchased from Lumiprobe (Alexa Fluor 647 and Cyanine5), Sigma (Atto655), Biotium (CF647, CF660C, and CF680), and Cytiva (Cy5.5). All these dyes were in the form of N-hydroxysuccinimidyl ester (NHS) for conjugation to secondary antibodies. The dye-to-antibody ratio was controlled to be less than one. Primary antibodies were obtained from ThermoFisher (rat antibody to alpha tubulin, MA1-80017; mouse antibody to beta tubulin, MA5-16308; goat antibody to HSPA9, PA5-48035; rabbit antibody to TOM20, MA5-34964) as gifts and purchased from Sigma (Chicken antibody to vimentin, AB5733; mouse antibody to PMP70, SAB4200181). Unconjugated secondary antibodies (Donkey anti-rat, 712-005-153; Donkey anti-mouse, 715-005-151; Donkey anti-rabbit, 711-005-152; Bovine anti-goat, 805-005-180) were purchased from Jackson ImmunoResearch Laboratories. For labelling actin, Alexa Fluor Plus 647 phalloidin were obtained from ThermoFisher as a gift.

S4. Sample preparation.

S4.1. Cell culture. COS-7, African green monkey kidney cells (CRL-1651, ATCC) was purchased from the American Type Culture Collection (ATCC). The COS-7 cells were cultured in T25 cell culture flask (690160, Greiner Bio-One) with the cell culture medium Dulbecco's Modified Eagle's Medium (DMEM) (10-014-CV, Corning) added with 10% fetal bovine serum (FBS) (26140079, Gibco) along with 1% Penicillin-Streptomycin (Pen Strep) (15140122, Gibco) (referred as the complete cell culture media in the rest of the article). µ-slide 8 Well high Glass Bottom chambered coverslip (80807, ibidi) was purchased from ibidi for subculturing cells. The chambers were rinsed with 1×PBS (10010023, Gibco) to remove glass dust before use. To subculture cells, to each well, 30 µL cell suspension solution was added. Then, 270 µL of complete cell culture media was added to every well. The chambered coverslip was kept in the cell culture incubator at 37°C with 5% CO_2 for 24 hours before the cells were used in imaging experiments. µ-dish (81158, ibidi) were also used in the imaging experiments. To subculture cells in µ-dish, 120 µL of cell suspension solution was added into the dish, followed by 2 mL of complete cell culture media. The cells were allowed to incubate for 24 hours at 37°C and 5% CO_2 inside the cell culture incubator before imaging.

S4.2. Cell labelling with far-red dyes. After 24 hours, COS-7 cells were washed once with pre-warmed 1×PBS buffer (10010049, ThermoFisher), fixed with pre-warmed 3% paraformaldehyde

(0433689M, ThermoFisher) + 0.1% glutaraldehyde (340855, Sigma) in 1×PBS buffer for 10 minutes, reduced with freshly prepared 0.3% $NaBH_4$ (BDH4604, VWR) for 10 minutes, followed by five-time washes with 1×PBS buffer. The cells were blocked and permeabilized in blocking buffer containing 3% BSA (001-000-162, Jackson ImmunoResearch Laboratories), 0.2% Triton-X100 (807426, MP Biomedicals) in 1×PBS buffer for 2 hours, followed by incubation with primary antibodies in blocking buffer at 4 ºC overnight. After primary antibody incubation, the cells were washed five times with washing buffer (0.75% BSA, 0.05% Triton-x100 in 1×PBS buffer), followed by incubation with dye-conjugated secondary antibodies in blocking buffer at room temperature for 2 hours. After secondary antibody incubation, the cells were washed five times with washing buffer, washed once with 1×PBS buffer, post-fixed with 3% paraformaldehyde and 0.1% glutaraldehyde in 1×PBS buffer for 10 minutes, followed by three-time wash with 1×PBS buffer. The cell sample was stored in 1×PBS buffer before microscopy imaging. For long-term storage, 20 mM (0.1%) sodium azide (14314.22, ThermoFisher) was added into the 1×PBS buffer. To co-stain actin with other subcellular structures, Alexa Fluor Plus 647 phalloidin (0.1 μM in 1×PBS buffer) was added to the cells after the post-fixation step, followed by incubation at 4 ºC overnight. The cells were briefly washed two times with 1×PBS buffer before imaging.

S5. SP-STORM imaging.

The dye labelled samples were first excited at low laser power for locating cells and capturing conventional fluorescence images. The laser power was then increased to high power density (628 nm, ~ 12 kW cm^{-2}) to photoswitch most of the dye molecules into nonfluorescent dark state. A random subset and spatially resolved dye molecules were kept in fluorescing (on state) at any given instant. 405 nm laser at low power density was used to adjust the density of single dye molecules per image frame when it is necessary. The EMCCD camera acquired images in all three channels (i.e., reference, sine- and cosine-modified) simultaneously and continuously at a frame rate of 200 Hz. The imaging data was typically recorded for 30-40k frames, which corresponds to an acquisition time of 2.5-3.3 minutes. SP-STORM imaging of far-red dyes were carried out in a buffer containing 10% (w/v) glucose, 0.5 mg/mL glucose oxidase, 40 μg/mL catalase, and 1% (v/v) beta-mercaptoethanol in 100 mM Tris-HCl buffer (pH 8.0).

S6. Data analysis.

The collected single molecule imaging data were analyzed by either ThunderStorm[2] ImageJ plugin or Insight3. The single molecules in all three channels were first identified and localized independently. To identify the same single molecule in all channels, a previously developed correlation analysis procedure was adapted.[1] We first imaged fluorescent beads on coverslip and use their localized positions to build a transformation matrix. The transformation matrix can then be used to project the spatial coordinates of localized single molecule positions from sine- and cosine-modified channel to the refence channel. The projected molecular positions are then

compared to those obtained in the refence channel. Molecular positions that are within the same imaging frame and within the localization precision are considered as from the same molecule. Their photon number were used for constructing phasor plot (G, S) and calculating the phase angle (φ) using the following equations:

$$G = 2\frac{(\frac{I_{cos}}{fI_0} - T_{\cos,min})}{(T_{\cos,max} - T_{cos,min})} - 1$$

$$S = 2\frac{(\frac{I_{sin}}{fI_0} - T_{\sin,min})}{(T_{\sin,max} - T_{sin,min})} - 1$$

$$\phi = \tan^{-1}(\frac{S}{G})$$

where I_0, I_{sin}, I_{cos} are photon number of single molecules in reference, sine, and cosine channels, respectively. f (~1.17) is the correction factor for the intrinsic difference in transmission efficiency between reference and sine/cosine channels. $T_{cos,max}$ and $T_{cos,min}$ are the maximum and minimum transmission of the cosine filter in the confined spectral window. $T_{sin,max}$ and $T_{sin,min}$ are the maximum and minimum transmission of the sine filter in the confined spectral window. Applying the calibration curve of spectral mean-phase angle relationships, we obtained the spectral mean (λ_{mean}). The final localized positions of single molecules were weighted average from those in all channels. Combining the obtained spectral mean and weighted average localized positions of single molecules, hyperspectral dSTORM image was rendered using Insight3 software. For classification of single molecules when the information of all dye molecules in the sample was known, boundary conditions in phasor plot were established from single-color labelled sample and used to separate the different dye molecules with a low crosstalk (Figure 4c, Figure S19g). Color crosstalk was then evaluated by applying these established boundary conditions to the results from single-color labeled sample. For 3D localizing single molecules' position, an elliptical 2D Gaussian function, $I(x,y) = hexp^{(-(2\frac{(x-x_0)^2}{w_x^2}+2\frac{(y-y_0)^2}{w_y^2}))} + b$, was used to fitting the imaging data. The obtained PSF widths (i.e., w_x and w_y) were then used to determine the z positions using the calibration curve (Figure S22). Post-data analysis of localized single molecules was done using MATLAB scripts and Insight3.

S7. Simulation study.

The emission spectra of used dyes in this work are broad. The locations of dyes in the spectral phasor plot depend on both the wavelength and the width of emission spectra, which lays the foundation for spectral demixing in phasor analysis. We use simulation results to demonstrate the working principle of spectral demixing based on phasor analysis and the effects of spectral properties on the performance. We simulated the fluorescent emission spectra at six peak

wavelengths (650-725 nm with 15 nm spectral interval) and four peak widths (full width at half maximum, FWHM= ~2.355σ: 10, 20, 50, and 100 nm) using the normalized Gaussian equation:

$$I(\lambda) = \exp\,(\frac{-(\lambda-\lambda_{peak})^2}{2\sigma^2})$$

where I(λ), λ_{peak}, and σ are intensity at λ, the peak wavelength, and standard deviation of the peak, respectively. The results are shown in Figure S2-3a.

S7.1. Using ideal sine/cosine transmission filters. Spectral phasor analysis using ideal sine/cosine filters were first conducted. The ideal sine/cosine transmission filters are simulated using the following equations:

$$T_{cos}(\lambda) = \cos\left[2\pi(\frac{\lambda}{\lambda_{max}-\lambda_{min}}+n)\right]$$

$$T_{sin}(\lambda) = \sin\left[2\pi(\frac{\lambda}{\lambda_{max}-\lambda_{min}}+n)\right]$$

$$n = 1-\frac{\lambda_{min}}{\lambda_{max}-\lambda_{min}}$$

where $T_{sin}(\lambda)$ and $T_{cos}(\lambda)$ are the transmission efficiency for sine and cosine filter at λ; λ_{min} and λ_{max} are the low and high end of detection window of wavelength; n is the correction factor that enables one period of sine/cosine wavefunction within the [λ_{min}, λ_{max}]. The simulated transmission profiles of ideal sine/cosine filters are shown in Figure S2. Applying the sine/cosine transmission filters on the simulated emission spectra transforms them into two modified emission spectra (Figure S2b-c). The phasor plot (G, S) can then be constructed using the following equation:

$$G = 2\frac{\int_{\lambda_{min}}^{\lambda_{max}} I(\lambda)\otimes T_{cos}(\lambda)d\lambda}{\int_{\lambda_{min}}^{\lambda_{max}} I(\lambda)d\lambda}-1$$

$$S = 2\frac{\int_{\lambda_{min}}^{\lambda_{max}} I(\lambda)\otimes T_{sin}(\lambda)d\lambda}{\int_{\lambda_{min}}^{\lambda_{max}} I(\lambda)d\lambda}-1$$

The results in spectra phasor plot for different peak wavelengths and widths are shown in Figure S2d. The results show two obvious trends: larger phase angles for emission at longer wavelengths and smaller phase amplitudes for wider emission spectra. In this work, the wavelength-phase angle relationship is of the most interest. Two types of wavelength-phase angle relationship were plotted using spectral peak and mean respectively (Figure S2e-f). Spectral mean was calculated as the intensity-weighted averaging of wavelength. Significant variation of peak wavelength-phase angle relationships was observed for wide emission spectra

(FWHM > 20 nm) due to the confined boundary of spectral detection window. On the contrary, the relationship between spectral mean and phase angle maintains very well (FWHM < 50 nm). In fact, phasor analysis was developed as a non-fitting method for analyzing spectral data and to obtain the spectral mean.

S7.2. Actual sin/cos filters. We further studied the effects of peak wavelength and width in the spectra phasor results when using the actual sine/cosine transmission filters in our experiments. Figure S3 shows the transmission profiles of the sine/cosine transmission filters measured by a UV-Vis spectrometer. Similarly, transformation of emission spectra with actual sine/cosine transmission filters can be done as that used with ideal sine/cosine transmission filters (Figure S3b-c). To construct the phasor plot (G, S), the following modified equations[3] were used to accommodate the min/max transmission of the actual sine/cosine transmission filters:

$$G = 2\frac{\frac{\int_{\lambda_{min}}^{\lambda_{max}} I(\lambda)\otimes T_{cos}(\lambda)d\lambda}{\int_{\lambda_{min}}^{\lambda_{max}} I(\lambda)d\lambda} - T_{cos,min}}{T_{cos,max} - T_{cos,min}} - 1$$

$$S = 2\frac{\frac{\int_{\lambda_{min}}^{\lambda_{max}} I(\lambda)\otimes T_{sin}(\lambda)d\lambda}{\int_{\lambda_{min}}^{\lambda_{max}} I(\lambda)d\lambda} - T_{sin,min}}{T_{sin,max} - T_{sin,min}} - 1$$

The results are shown in Figure S3d. A nonlinear relationship between wavelength (either spectral peak or spectral mean) and phase angle were obtained (Figure S3e-f), which matches with the calibration results using mechanical slits and fluorescent beads (Figure 1e). The same results were also observed for the variation of wavelength-phase angle relationships that spectral mean-phase angle relationship maintains the trend for emission spectra with FWHM < 50 nm. In this work, the FWHMs of tested far-red dyes are less than 50 nm.

S8. Supporting Figures.

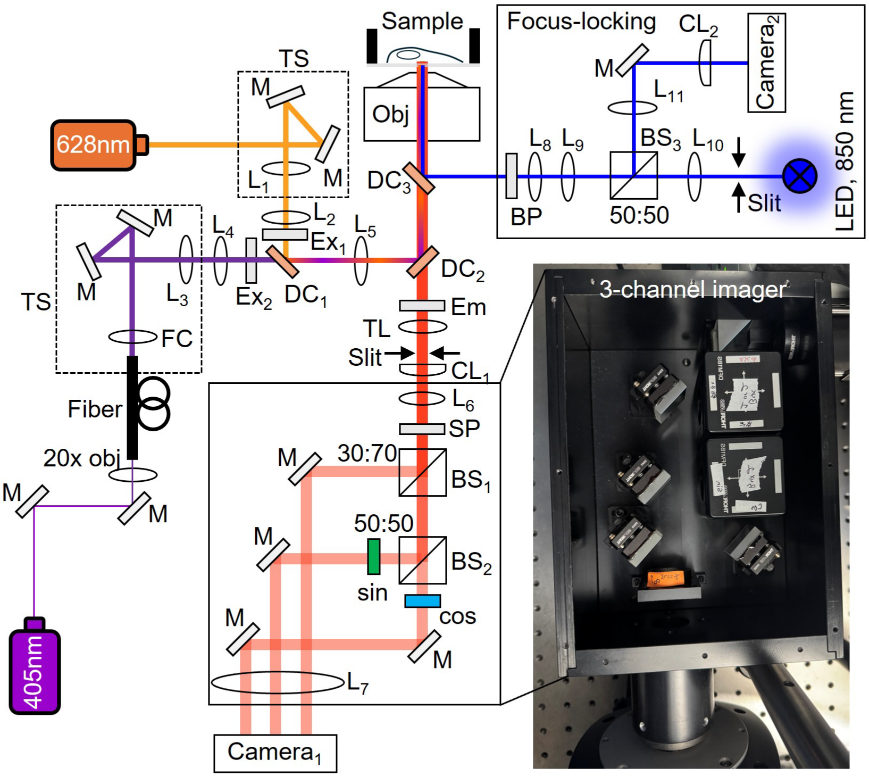

Figure S1. Optical setup of SP-STORM. The imaging system is built with an Olympus IX81 motorized inverted microscope.

Light sources: Lasers, 100 mW 405 nm (Coherent Obis) and 200 mW 628 nm (MPB communications); LED, 1400 mW 850nm (M850LP1, Thorlabs).

Optics: M, BB1-E02 or BBSQ1-E02 or MRA25-E02 (Thorlabs); Lens1-7, AC254-075-A, AC254-100-A, AC254-100-A, AC254-100-A, AC254-200-A, AC254-125-A, AC254-125-A (Thorlabs); Lens 8-11, LB1945-AB, LB1676-AB, LB1757-AB, LB1901-AB (Thorlabs); CL_1, LJ1516RM-A, CL_2, LJ1703RM-B (Thorlabs); DC1, 69-204 (Edmund Optics); DC2, RT633rdc (Chroma); DC3, FF750-SDi02 (Semrocks); Ex1, RET633/5x (Chroma); Ex2, ZET405/488/561/640xv2 (Chroma); Em, RET638lp (Chroma); SP, FESH0750 (Thorlabs); BS1, BS019 (Thorlabs); BS2, BS013 (Thorlabs); BS3, BS014 (Thorlabs); BP, FF01-857/30 (Semrock); sine/cosine filters, 700FS80/650FS80 (Andover).

Objectives: 20× air objective (Olympus); 100× Oil TIRF objective (UPLAPO100XOHR, N.A. 1.50, Olympus)

Fiber components: FC, TC18APC-543 (Thorlabs); fiber, P3-S405-FC-1 (Thorlabs).

Mechanic and motorized components: TS, PT1-Z9 and KDC101 (Thorlabs); slit, VA100CP (Thorlabs).

Detectors: camera 1, Andor iXonEM+ Ultra 897; camera 2, DCC1545M (Thorlabs).

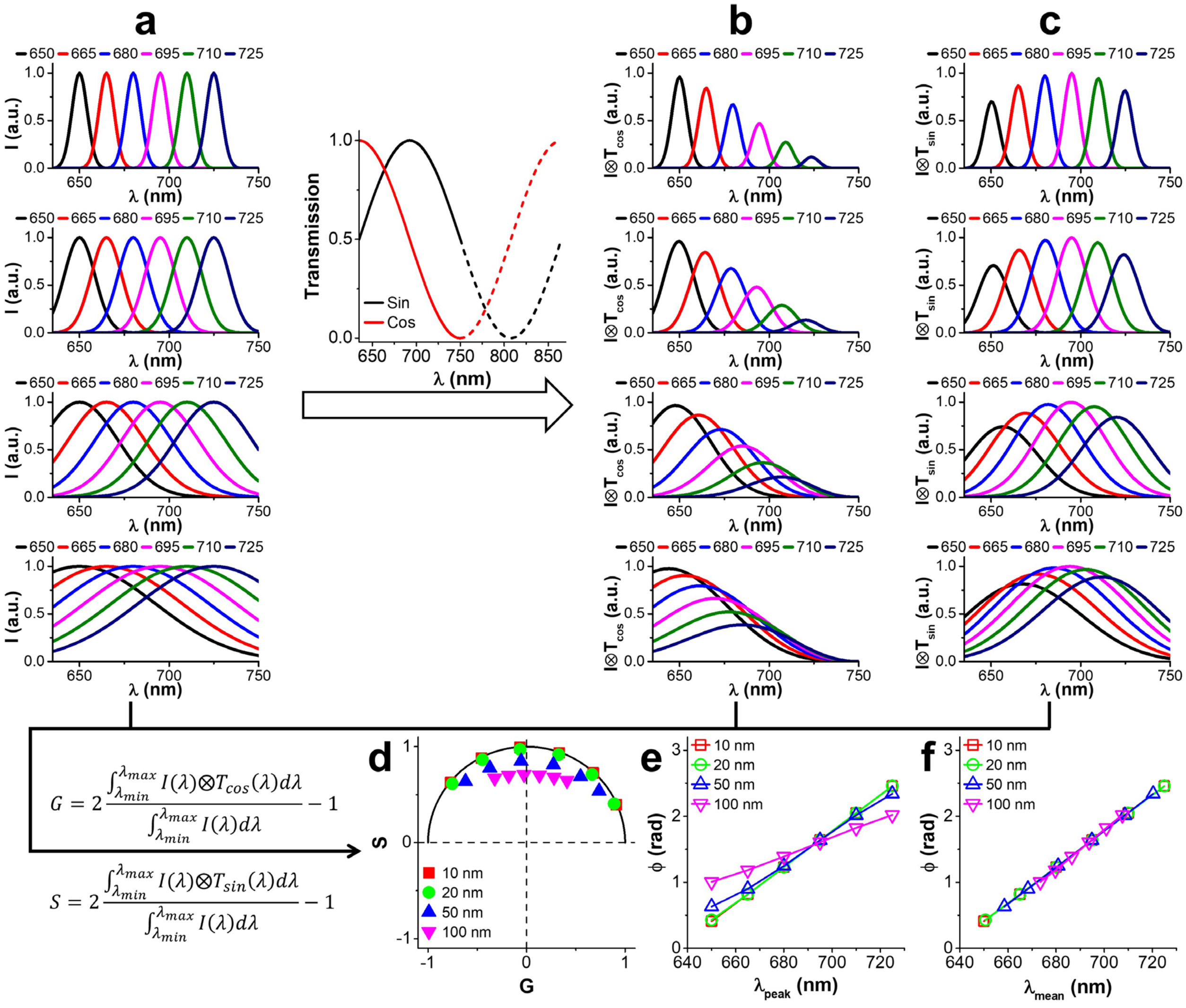

Figure S2. Spectral phasor analysis with ideal sine and cosine transmission filters for far-red dyes. (a) Simulated emission spectra with Gaussian distribution of different full width of half maximum. (b) Cosine and (c) sine modified emission spectra from (a). (d) Phasor plot. Black solid line denotes results directly calculated from transmission profiles of ideal sine and cosine filters, representing phasor plot at single-wavelength resolution. (e) phase angle-peak wavelength and (f) phase angle-spectral mean relationships, respectively.

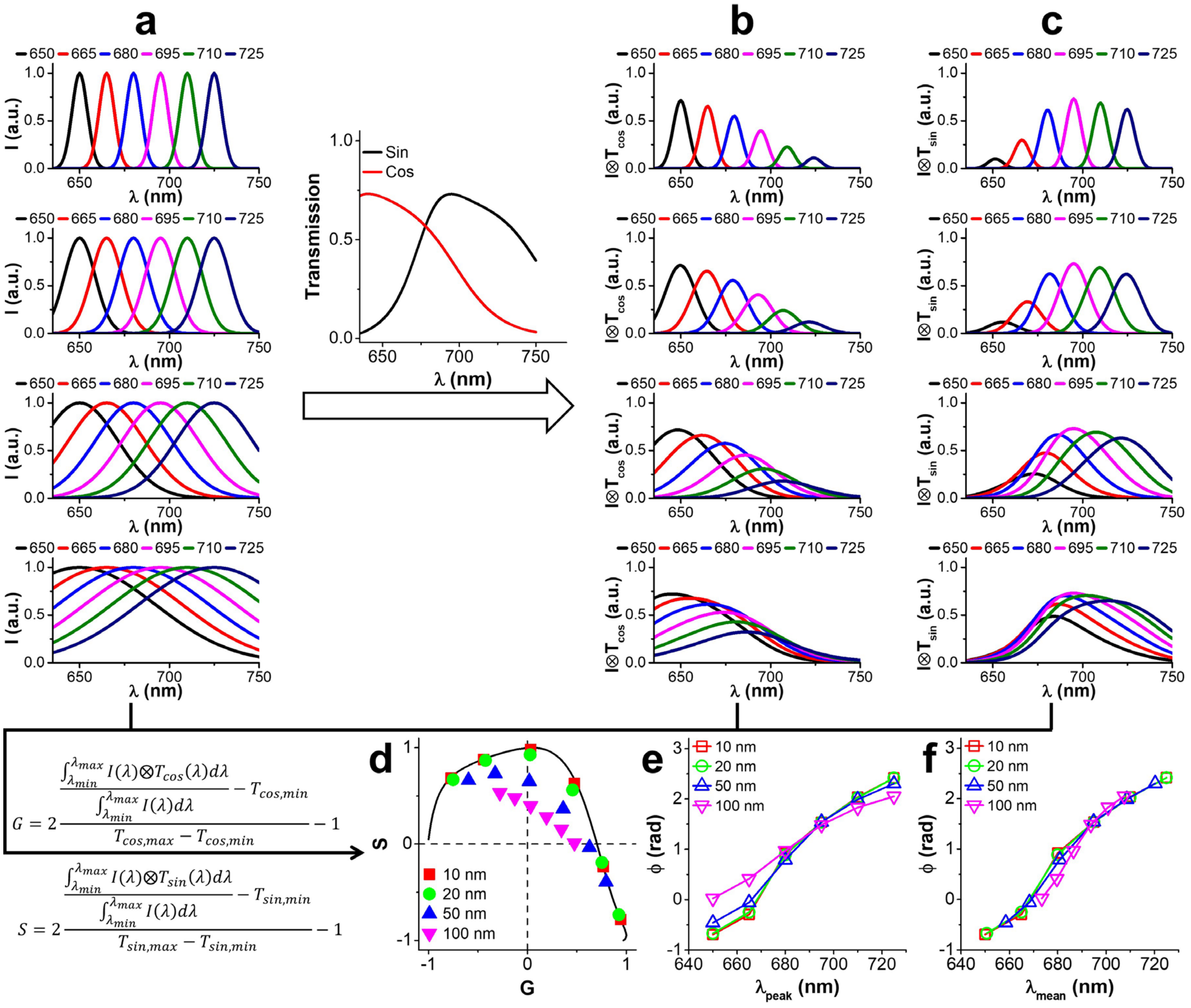

Figure S3. Spectral phasor analysis with actual sine and cosine filters for far-red dyes. (a) Simulated emission spectra with Gaussian distribution of different full width of half maximum. (b) Cosine and (c) sine modified emission spectra from (a). (d) Phasor plot. Black solid line denotes results directly calculated from transmission profiles of actual sine and cosine filters, representing phasor plot at single-wavelength resolution. (e) phase angle-peak wavelength and (f) phase angle-spectral mean relationships, respectively.

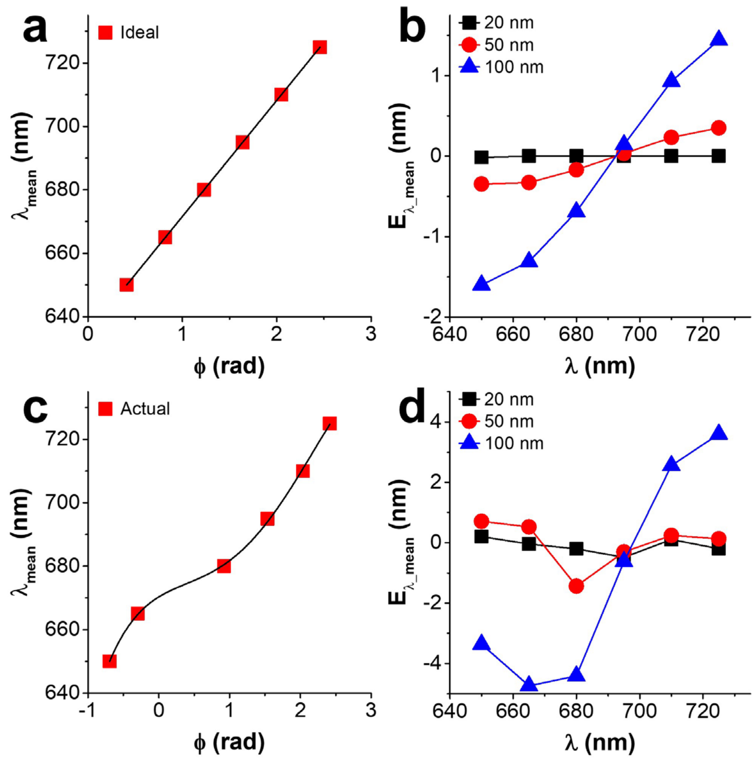

Figure S4. Error evaluation of spectral mean. (a, c) The calibration curve for spectral mean-phase angle relationship from simulation data with10 nm FWHM, similarly to the obtained calibration curve experimentally (Figure 1b-e). (b, d) The errors, defined as the difference between the spectral mean determined from the calibration curve and the weighted spectral mean from ground truth, under different FWHMs and at different peak wavelengths.

Using simulation data, we here evaluated the impact of using nonideal optical filters on the error in determined spectral results with different peak bandwidths and at different peak wavelengths. The results indicate that larger spectral errors were obtained with increased peak width, when peak wavelength is located near the boundary of spectral window of detection, and when using nonideal filters than the ideal ones. However, the error in spectral color was estimated to be smaller than 1 nm for spectral with peak width below 50 nm. The bandwidths of all tested organic dyes in this work are smaller than 50 nm suggesting the error in the spectral results from using nonideal optical filters is minimum.

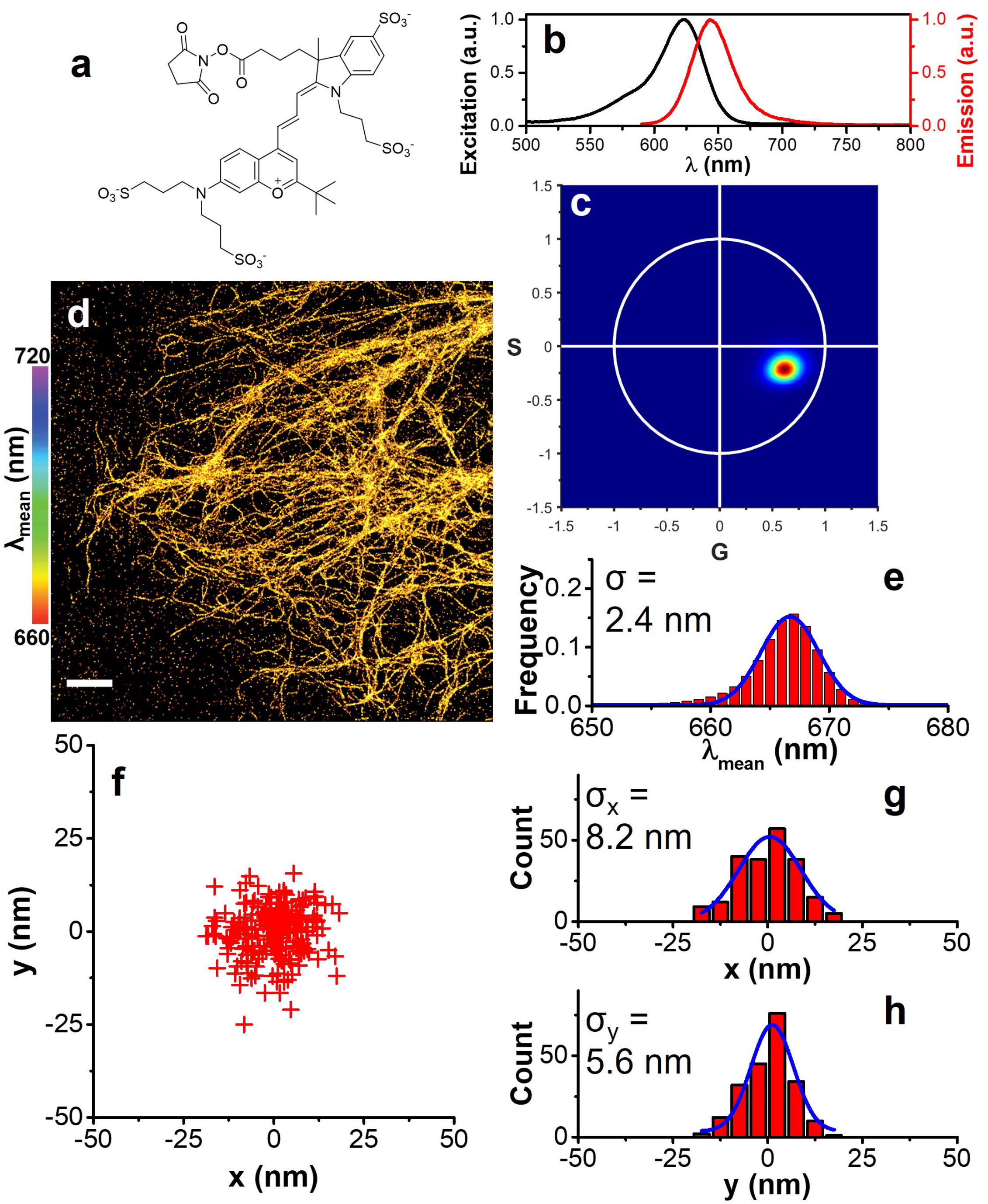

Figure S5. Evaluation of DyLight633 dye for SP-STORM. (a) Chemical structure. The structure was obtained from the manufacturer. (b) Excitation and emission spectra. (c) Phasor plot of >10^5 single DL633 molecules. (d) Hyperspectral dSTORM image of vimentin labelled by DL633 in fixed COS-7 cells. (e) 1D Gaussian fitting the histogram of the spectral mean of single DL633 molecules gives an average of 666.7 ± 2.4 nm (mean ± s.d.). (f) Cluster analysis of locations. (g-h) Fitting histogram distributions in x, y gives standard deviation of σ_x = 8.2 and σ_y = 5.6 nm respectively. Scale bar: 2 μm.

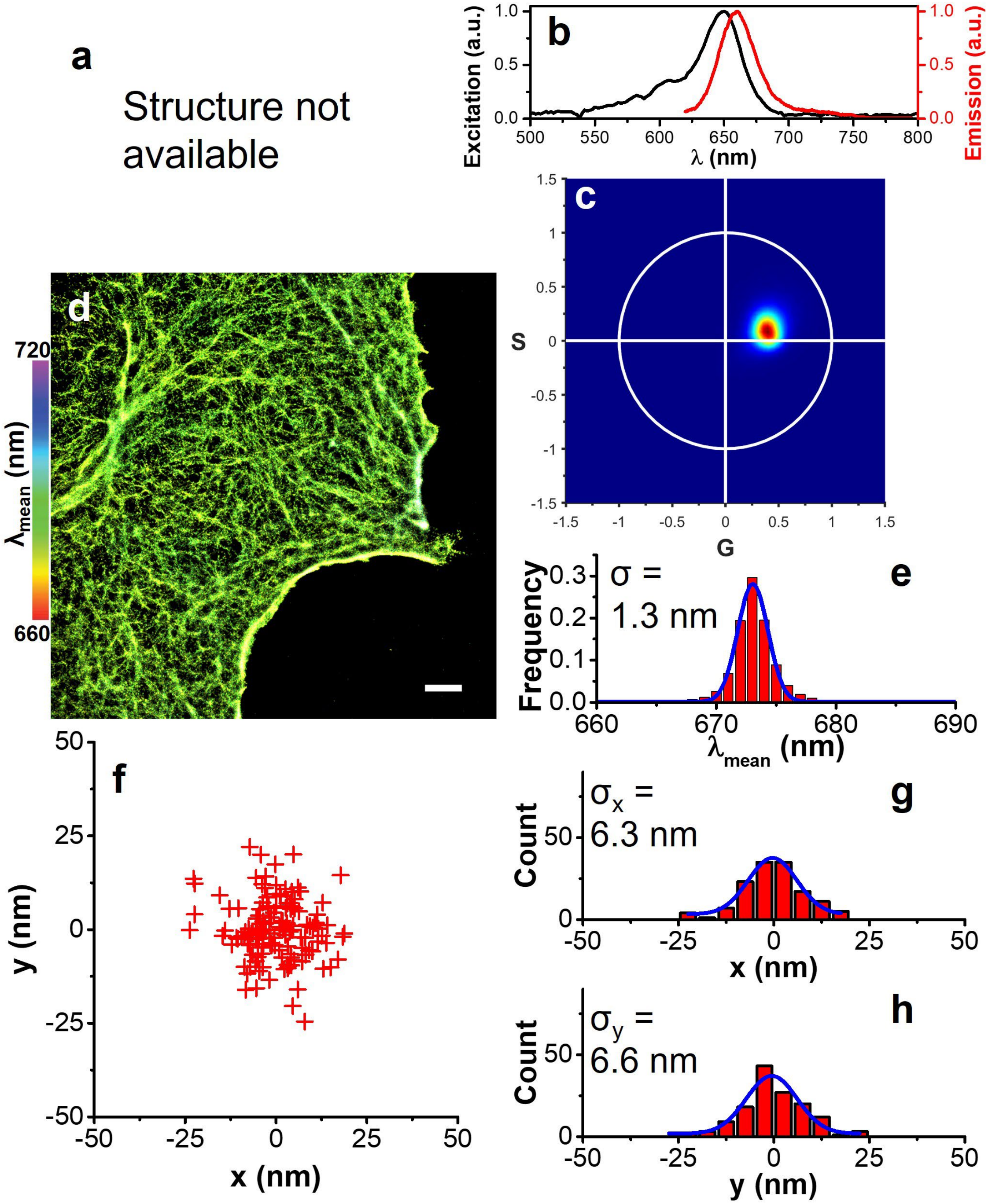

Figure S6. Evaluation of Alexa fluor plus 647 dye for SP-STORM. (a) Chemical structure is not available. (b) Excitation and emission spectra. (c) Phasor plot of $>10^5$ single $AF647^+$ molecules. (d) Hyperspectral dSTORM image of F-actin labelled by $AF647^+$ in fixed COS-7 cells. (e) 1D Gaussian fitting the histogram of the spectral mean of single $AF647^+$ molecules gives an average of 672.7 ± 1.3 nm (mean ± s.d.). (f) Cluster analysis of locations. (g-h) Fitting histogram distributions in x, y gives standard deviation of $\sigma_x = 6.3$ and $\sigma_y = 6.6$ nm respectively. Scale bar: 2 μm.

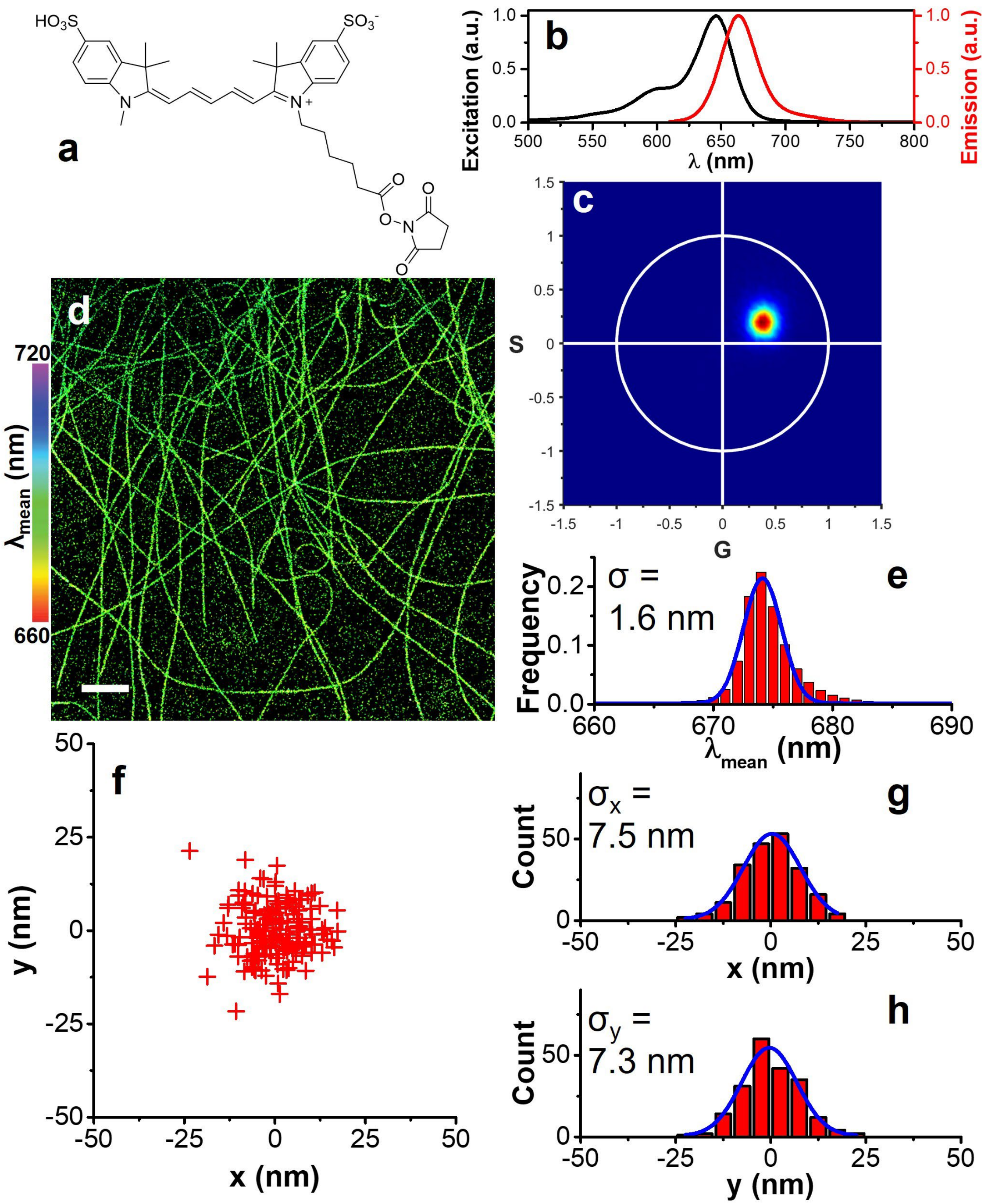

Figure S7. Evaluation of sulfo-cyanine5 dye lumiprobe for SP-STORM. (a) Chemical structure. The structure was obtained from the manufacturer. (b) Excitation and emission spectra. (c) Phasor plot of $>10^5$ single Cy5 molecules. (d) Hyperspectral dSTORM image of α-tubulin labelled by Cy5 in fixed COS-7 cells. (e) 1D Gaussian fitting the histogram of the spectral mean of single Cy5 molecules gives an average of 674.1 ± 1.6 nm (mean ± s.d.). (f) Cluster analysis of locations. (g-h) Fitting histogram distributions in x, y gives standard deviation of $\sigma_x = 7.5$ and $\sigma_y = 7.3$ nm respectively. Scale bar: 2 μm.

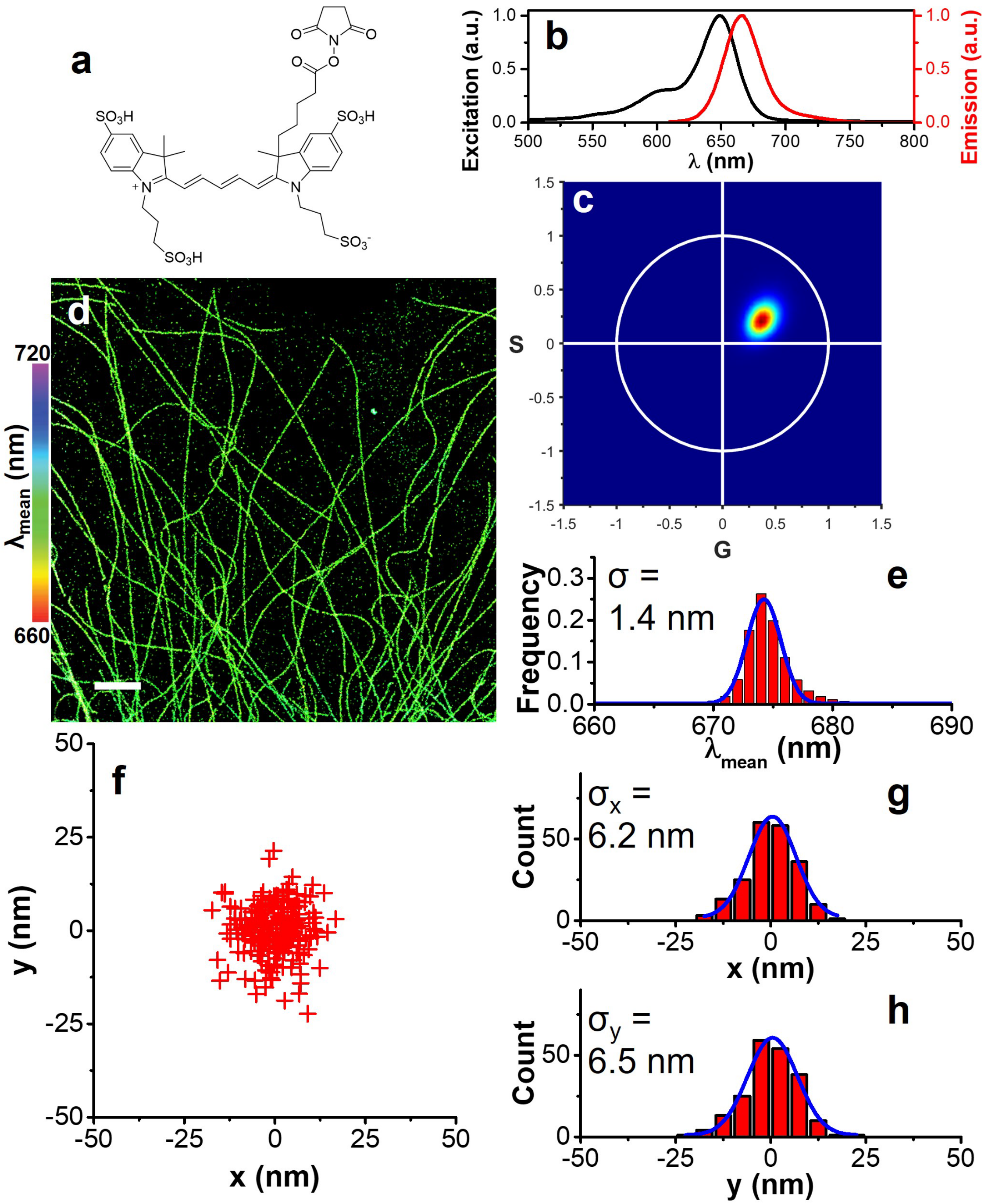

Figure S8. Evaluation of Alexa fluor 647 dye for SP-STORM. (a) Chemical structure. The structure was obtained from the literature.[4] (b) Excitation and emission spectra. (c) Phasor plot of >10^5 single AF647 molecules. (d) Hyperspectral dSTORM image of α-tubulin labelled by AF647 in fixed COS-7 cells. (e) 1D Gaussian fitting the histogram of the spectral mean of single AF647 molecules gives an average of 674.2 ± 1.4 nm (mean ± s.d.). (f) Cluster analysis of locations. (g-h) Fitting histogram distributions in x, y gives standard deviation of σ_x = 6.2 and σ_y = 6.5 nm respectively. Scale bar: 2 μm.

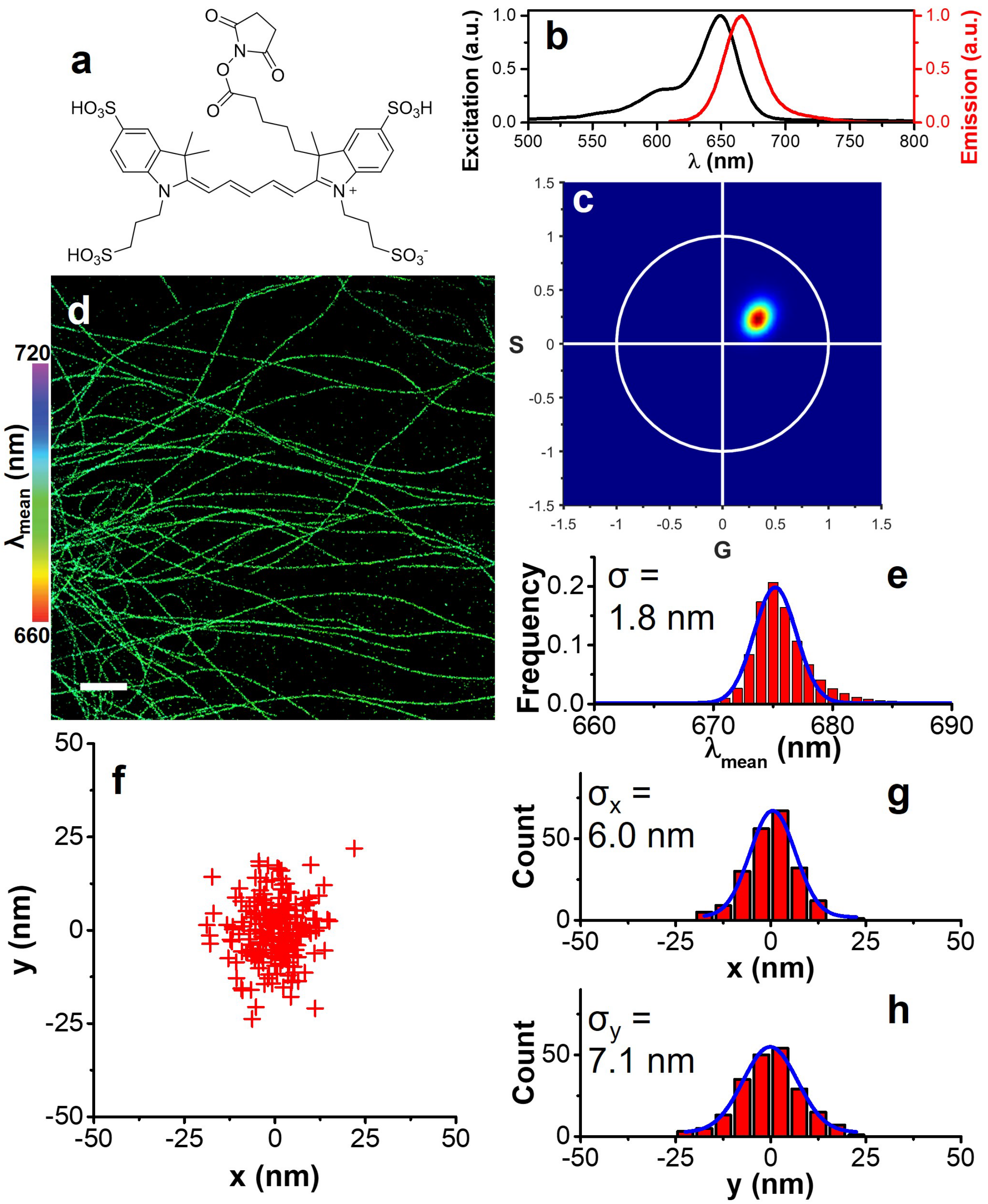

Figure S9. Evaluation of Alexa fluor 647 dye from Lumiprobe for SP-STORM. (a) Chemical structure. The structure was obtained from the manufacturer. (b) Excitation and emission spectra. (c) Phasor plot of $>10^5$ single AF647^{lumi} molecules. (d) Hyperspectral dSTORM image of α-tubulin labelled by AF647^{lumi} in fixed COS-7 cells. (e) 1D Gaussian fitting the histogram of the spectral mean of single AF647^{lumi} molecules gives an average of 675.2 ± 1.8 nm (mean ± s.d.). (f) Cluster analysis of locations. (g-h) Fitting histogram distributions in x, y gives standard deviation of $σ_x$ = 6.0 and $σ_y$ = 7.1 nm respectively. Scale bar: 2 μm.

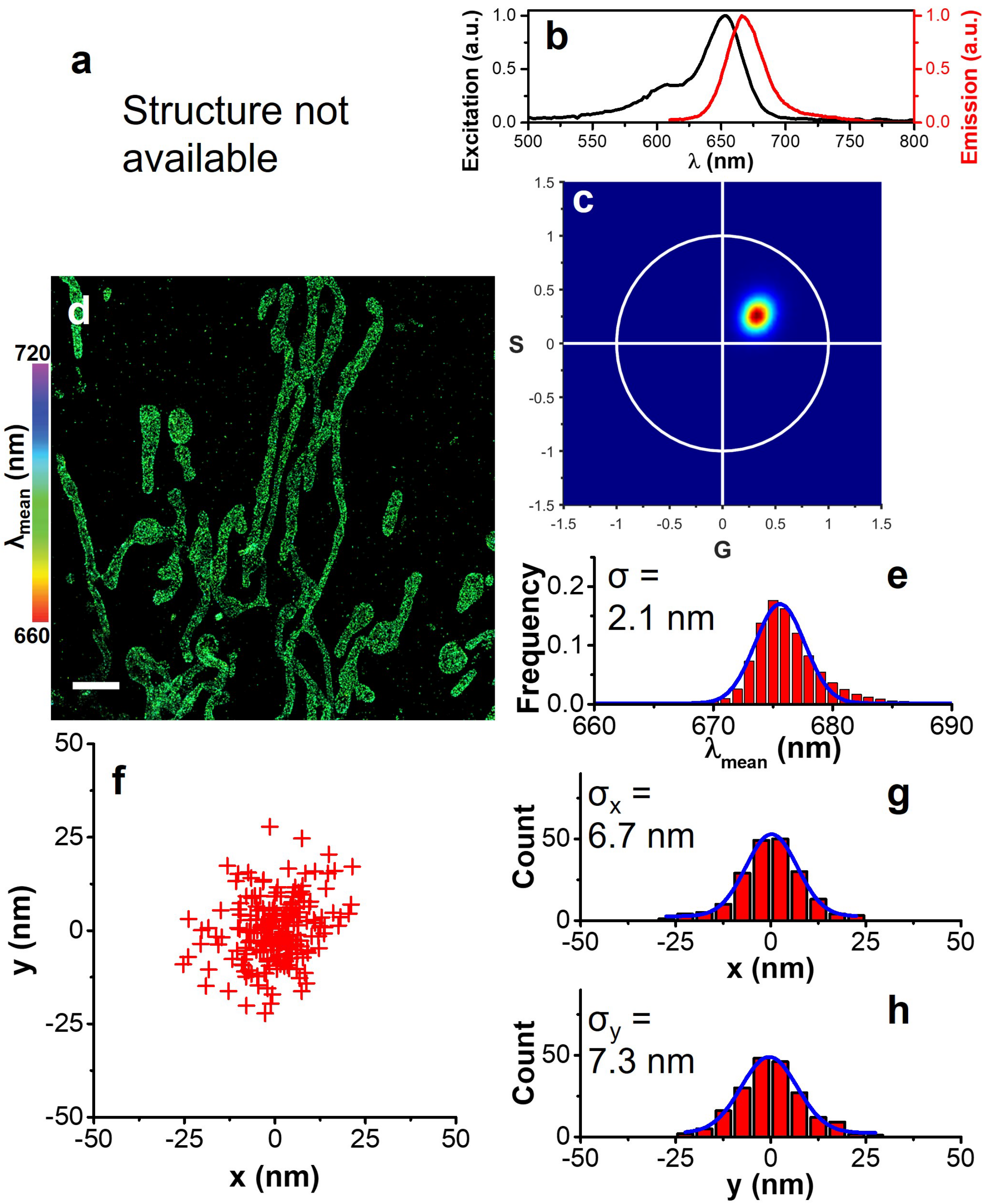

Figure S10. Evaluation of CF647 dye for SP-STORM. (a) Chemical structure is not available. (b) Excitation and emission spectra. (c) Phasor plot of >10^5 single CF647 molecules. (d) Hyperspectral dSTORM image of TOM20 labelled by CF647 in fixed COS-7 cells. (e) 1D Gaussian fitting the histogram of the spectral mean of single CF647 molecules gives an average of 675.6 ± 2.1 nm (mean ± s.d.). (f) Cluster analysis of locations. (g-h) Fitting histogram distributions in x, y gives standard deviation of σ_x = 6.7 and σ_y = 7.3 nm respectively. Scale bar: 2 μm.

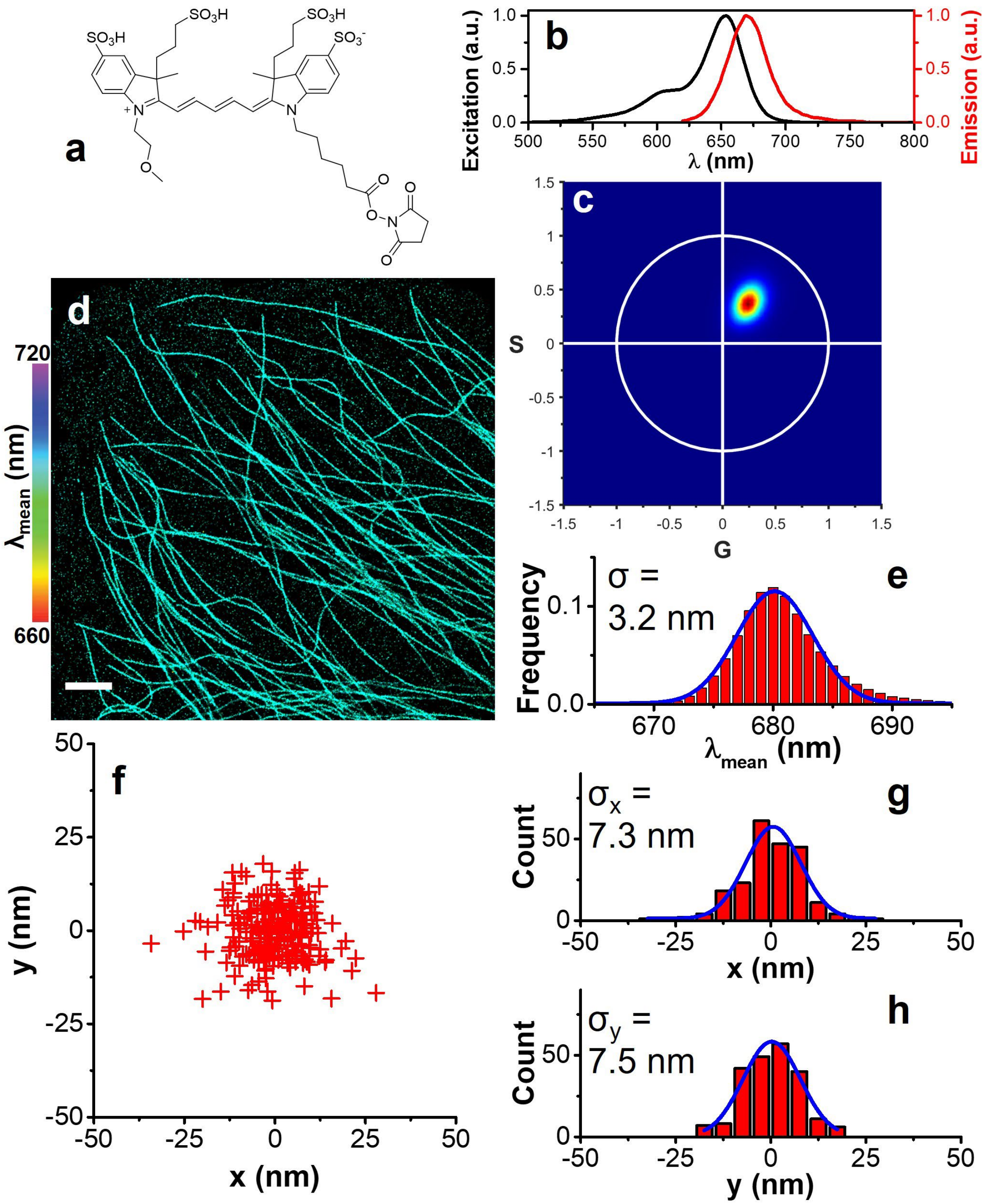

Figure S11. Evaluation of DyLight650 dye for SP-STORM. (a) Chemical structure. The structure was obtained from the manufacturer. (b) Excitation and emission spectra. (c) Phasor plot of >10^5 single DL650 molecules. (d) Hyperspectral dSTORM image of α-tubulin labelled by DL650 in fixed COS-7 cells. (e) 1D Gaussian fitting the histogram of the spectral mean of single DL650 molecules gives an average of 680.1 ± 3.2 nm (mean ± s.d.). (f) Cluster analysis of locations. (g-h) Fitting histogram distributions in x, y gives standard deviation of σ_x = 7.3 and σ_y = 7.5 nm respectively. Scale bar: 2 µm.

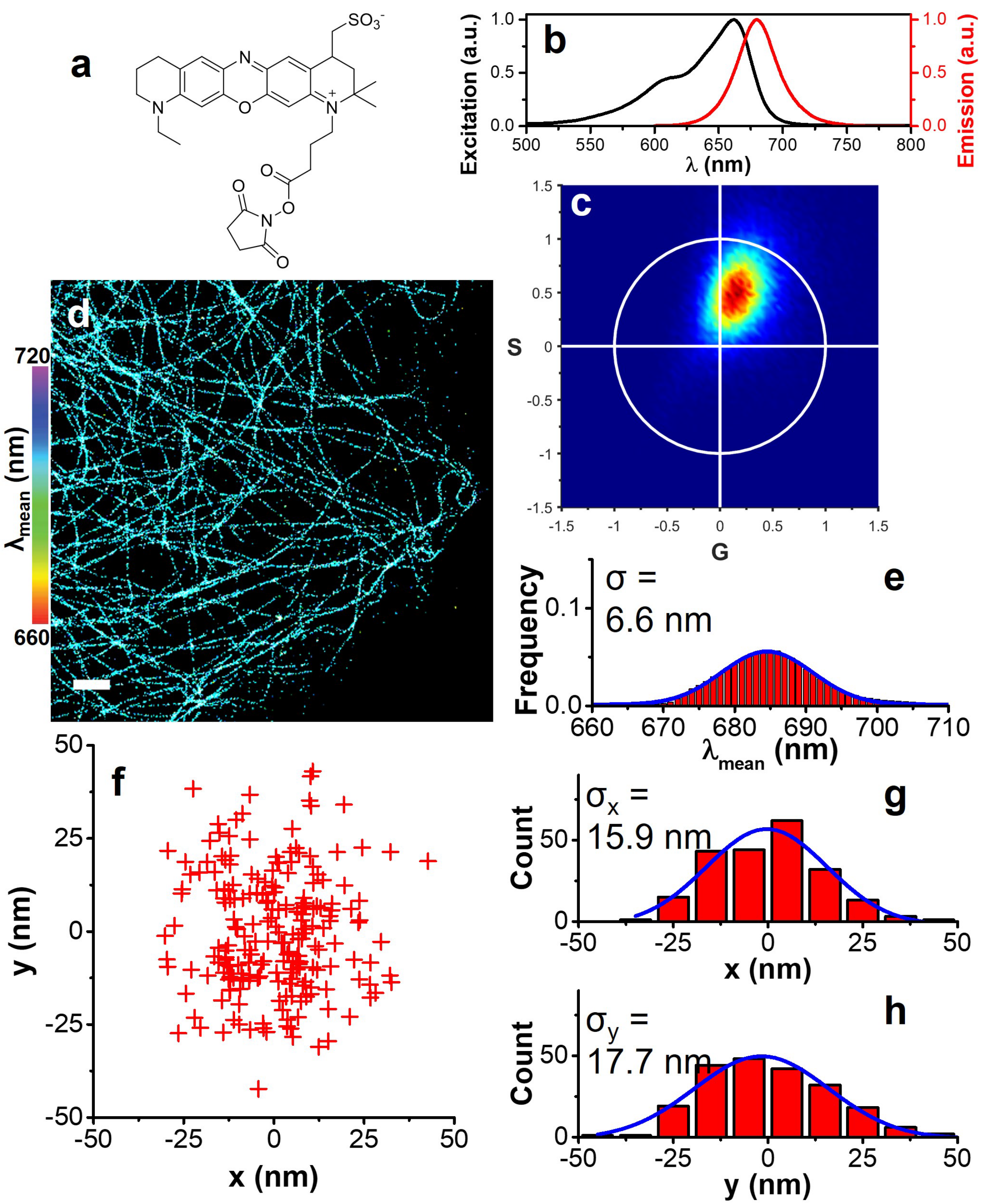

Figure S12. Evaluation of Atto655 dye for SP-STORM. (a) Chemical structure. The structure was obtained from the literature.[4] (b) Excitation and emission spectra. (c) Phasor plot of $>10^5$ single Atto655 molecules. (d) Hyperspectral dSTORM image of β-tubulin labelled by Atto655 in fixed COS-7 cells. (e) 1D Gaussian fitting the histogram of the spectral mean of single Atto655 molecules gives an average of 684.6 ± 6.6 nm (mean ± s.d.). (f) Cluster analysis of locations. (g-h) Fitting histogram distributions in x, y gives standard deviation of $\sigma_x = 15.9$ and $\sigma_y = 17.7$ nm respectively. Scale bar: 2 μm.

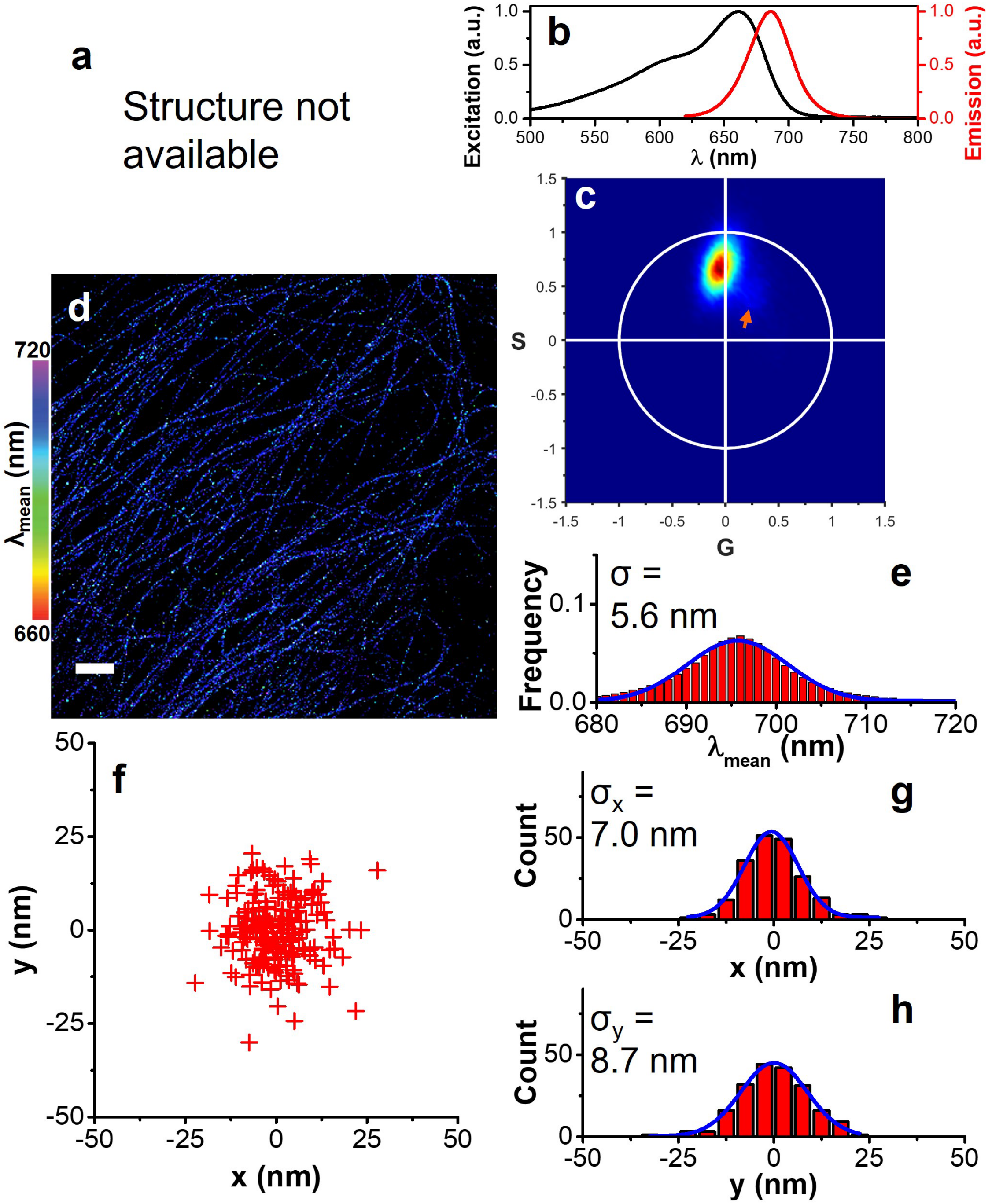

Figure S13. Evaluation of Alexa fluor 660 dye for SP-STORM. (a) Chemical structure is not available. (b) Excitation and emission spectra. (c) Phasor plot of >10^5 single AF660 molecules. A sub-population of smaller phase angles was identified (highlighted by orange arrow). (d) Hyperspectral dSTORM image of β-tubulin labelled by AF660 in fixed COS-7 cells. (e) 1D Gaussian fitting the histogram of the spectral mean of single AF660 molecules gives an average of 695.7 ± 5.6 nm (mean ± s.d.). (f) Cluster analysis of locations. (g-h) Fitting histogram distributions in x, y gives standard deviation of σ_x = 7.0 and σ_y = 8.7 nm respectively. Scale bar: 2 μm.

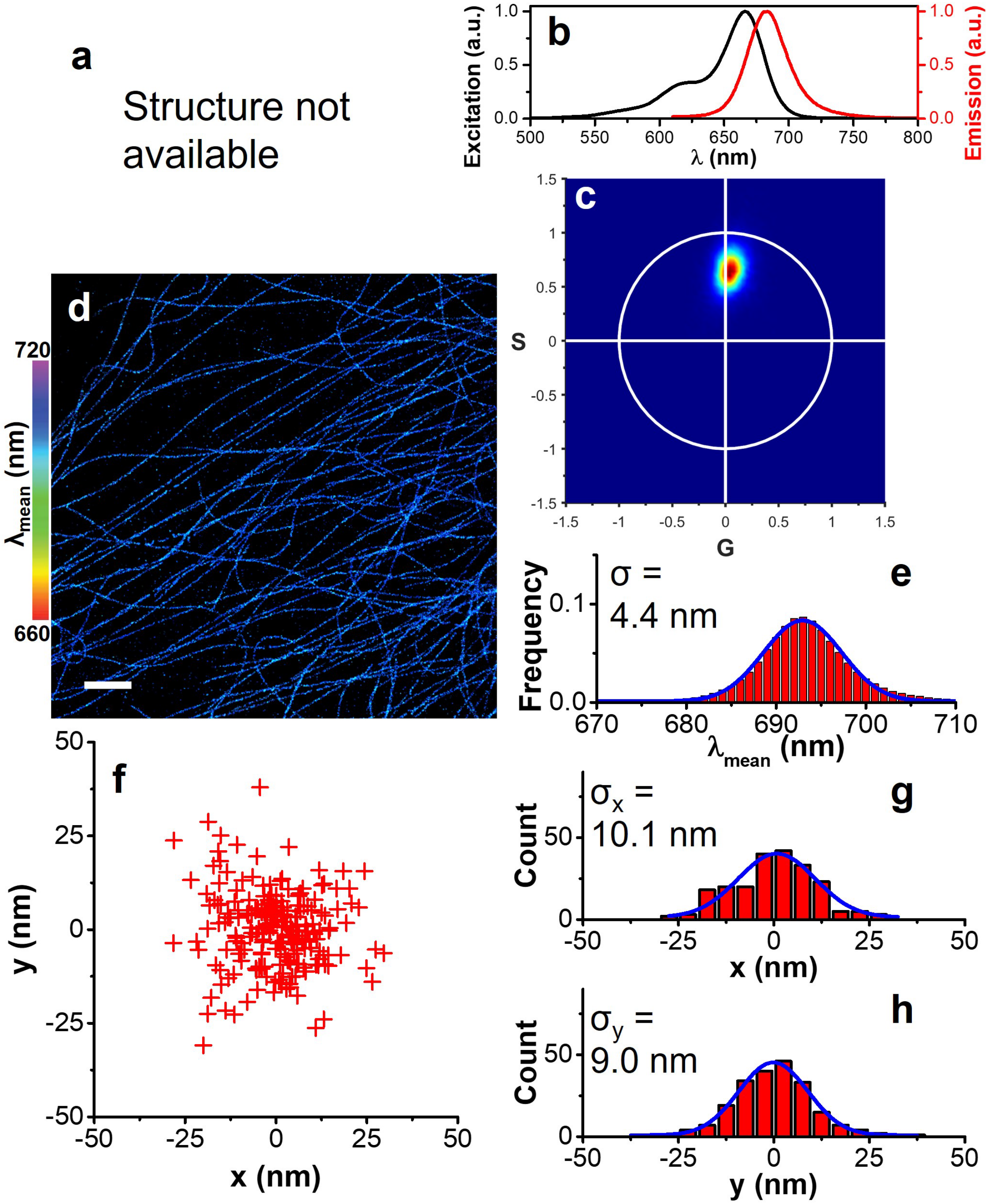

Figure S14. Evaluation of CF660C dye for SP-STORM. (a) Chemical structure is not available. (b) Excitation and emission spectra. (c) Phasor plot of >10^5 single CF660C molecules. (d) Hyperspectral dSTORM image of α-tubulin labelled by CF660C in fixed COS-7 cells. (e) 1D Gaussian fitting the histogram of the spectral mean of single CF660C molecules gives an average of 692.9 ± 4.4 nm (mean ± s.d.). (f) Cluster analysis of locations. (g-h) Fitting histogram distributions in x, y gives standard deviation of σ_x = 10.1 and σ_y = 9.0 nm respectively. Scale bar: 2 μm.

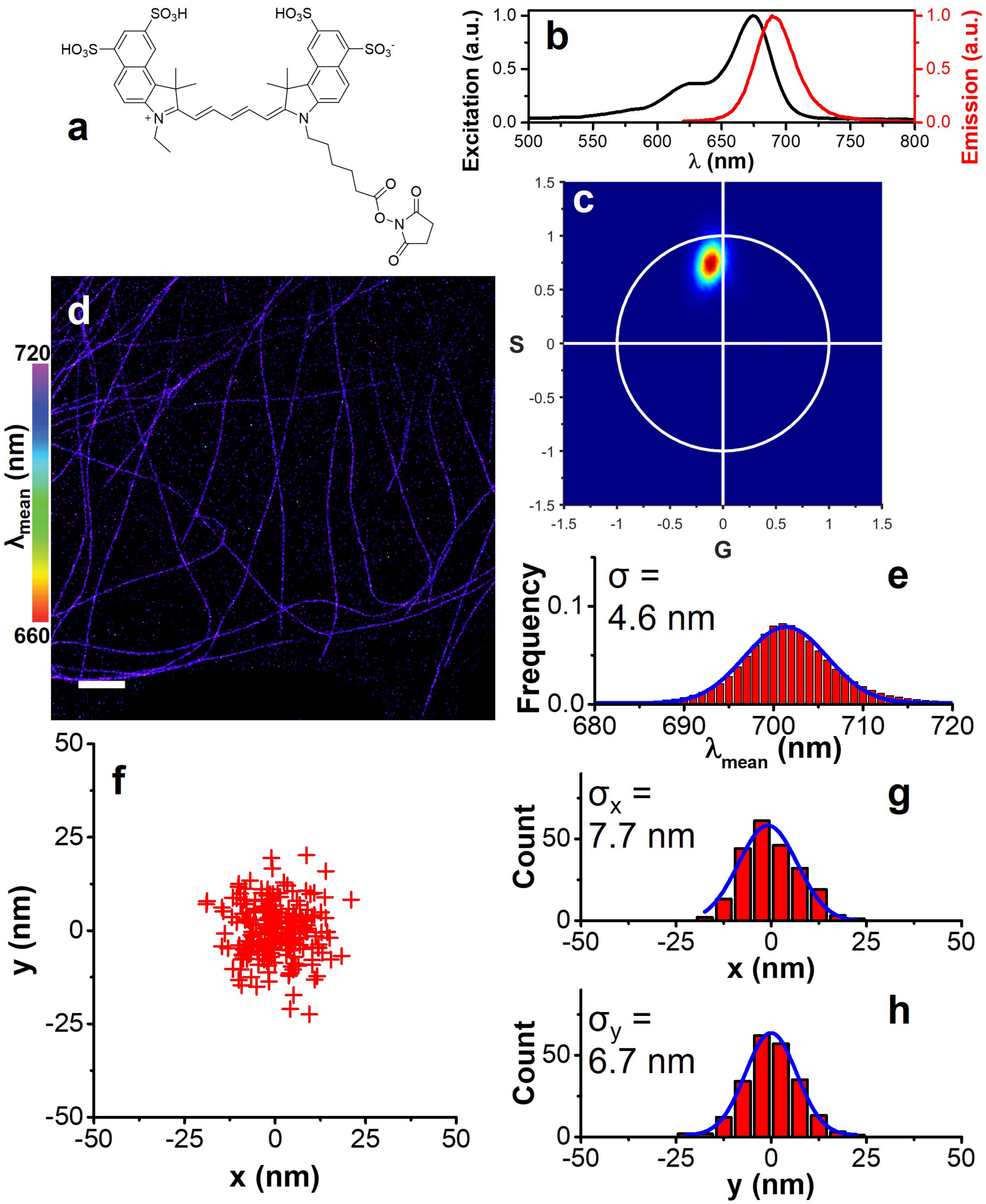

Figure S15. Evaluation of Cyanine5.5 dye for SP-STORM. (a) Chemical structure. The structure was obtained from the manufacturer. (b) Excitation and emission spectra. (c) Phasor plot of >10^5 single Cy5.5 molecules. (d) Hyperspectral dSTORM image of α-tubulin labelled by Cy5.5 in fixed COS-7 cells. (e) 1D Gaussian fitting the histogram of the spectral mean of single Cy5.5 molecules gives an average of 701.4 ± 4.6 nm (mean ± s.d.). (f) Cluster analysis of locations. (g-h) Fitting histogram distributions in x, y gives standard deviation of σ_x = 7.7 and σ_y = 6.7 nm respectively. Scale bar: 2 μm.

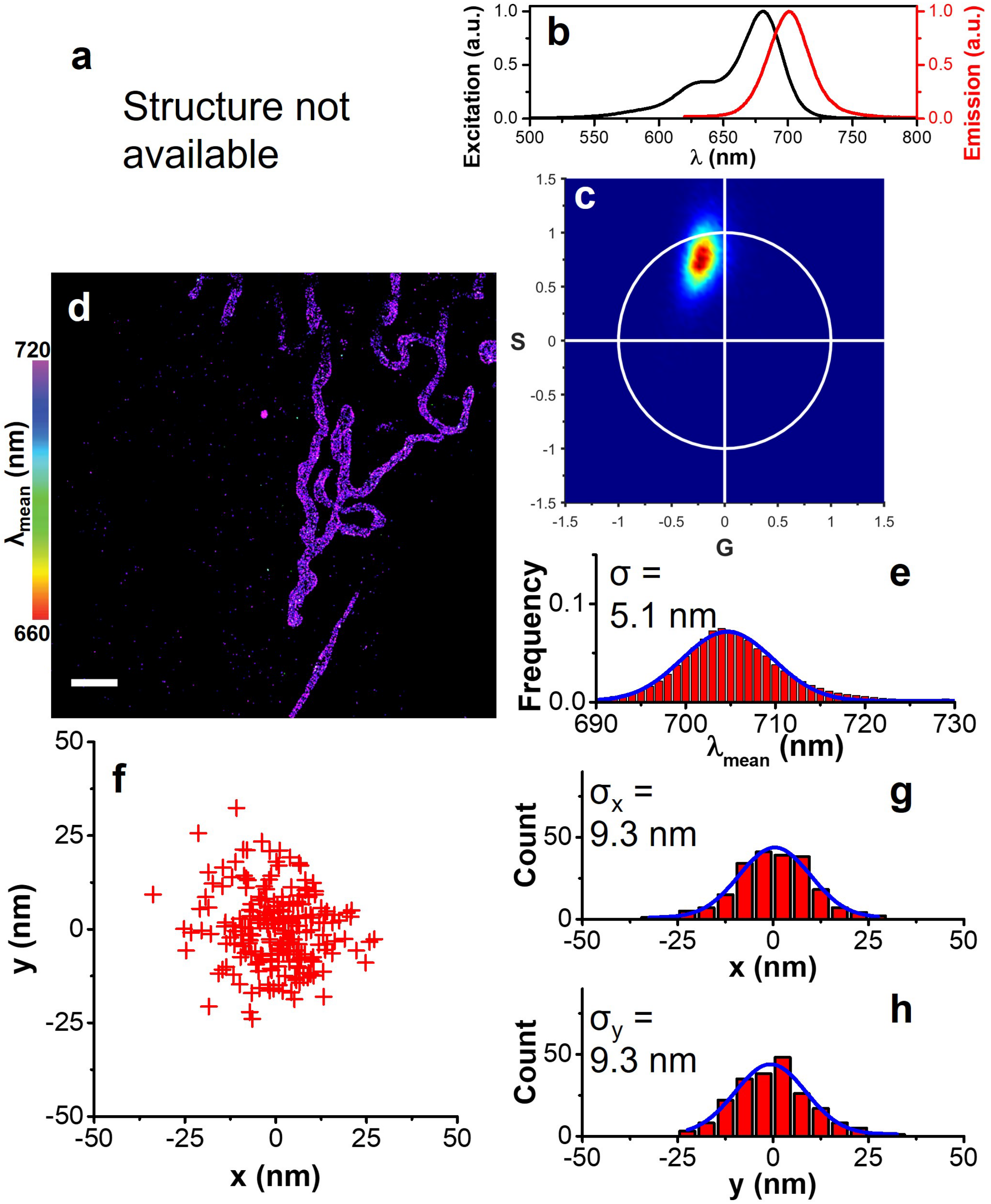

Figure S16. Evaluation of CF680 dye for SP-STORM. (a) Chemical structure is not available. (b) Excitation and emission spectra. (c) Phasor plot of >10^5 single CF680 molecules. (d) Hyperspectral dSTORM image of TOM20 labelled by CF680 in fixed COS-7 cells. (e) 1D Gaussian fitting the histogram of the spectral mean of single CF680 molecules gives an average of 704.6 ± 5.1 nm (mean ± s.d.). (f) Cluster analysis of locations. (g-h) Fitting histogram distributions in x, y gives standard deviation of σ_x = 9.3 and σ_y = 9.3 nm respectively. Scale bar: 2 μm.

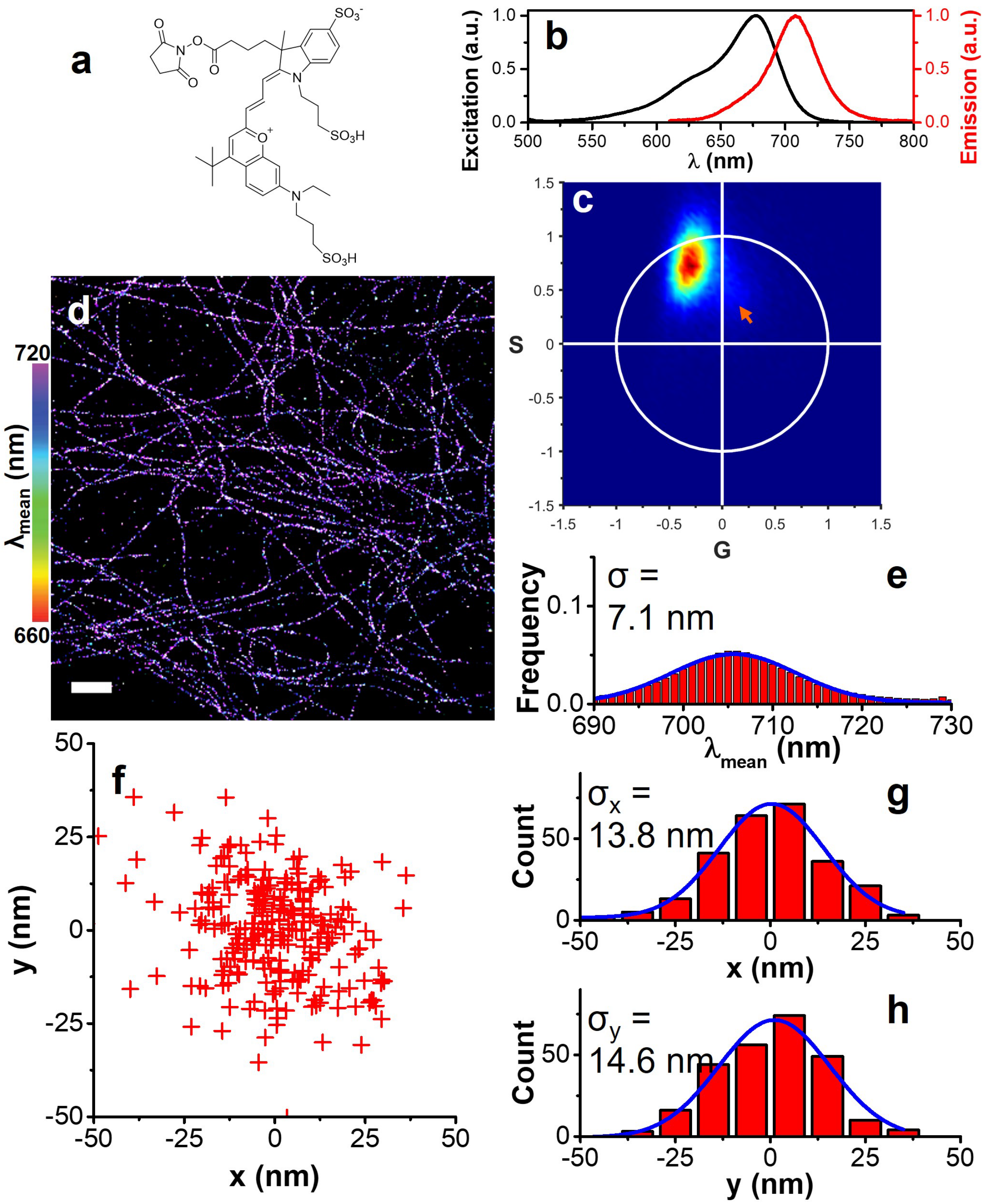

Figure S17. Evaluation of DyLight680 dye for SP-STORM. (a) Chemical structure. The structure was obtained from the manufacturer. (b) Excitation and emission spectra. (c) Phasor plot of >10^5 single DL680 molecules. A sub-population of smaller phase angles was identified (highlighted by orange arrow). (d) Hyperspectral dSTORM image of β-tubulin labelled by DL680 in fixed COS-7 cells. (e) 1D Gaussian fitting the histogram of the spectral mean of single DL680 molecules gives an average of 705.7 ± 7.1 nm (mean ± s.d.). (f) Cluster analysis of locations. (g-h) Fitting histogram distributions in x, y gives standard deviation of σ_x = 13.8 and σ_y = 14.6 nm respectively. Scale bar: 2 μm.

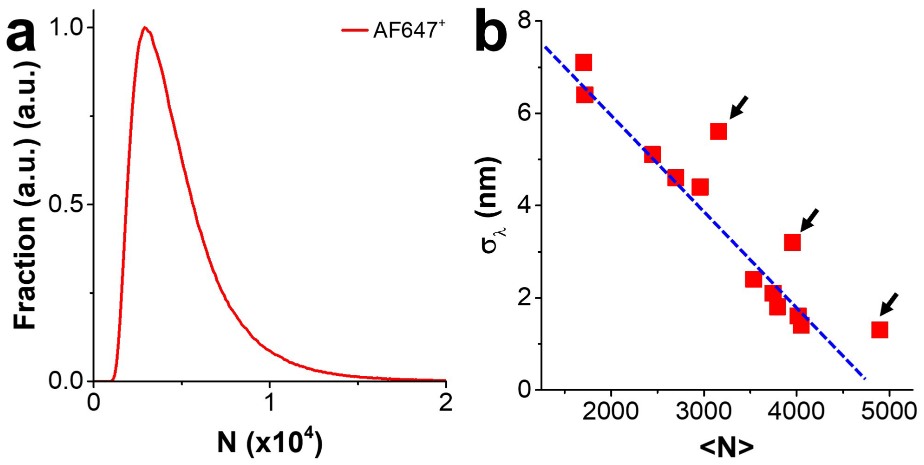

Figure S18. The dependence of spectral variation on photon number. (a) Probability distribution of photon number collected from single AF plus 647 dye molecules per on-off cycle. (b) Relationship between single-molecule spectral variation and photon number. The blue dash line was drawn as a guideline of the general trend. Black arrow highlights the cases that larger photon number does not always result in smaller spectral variation due to inherent heterogeneity at single molecule level.

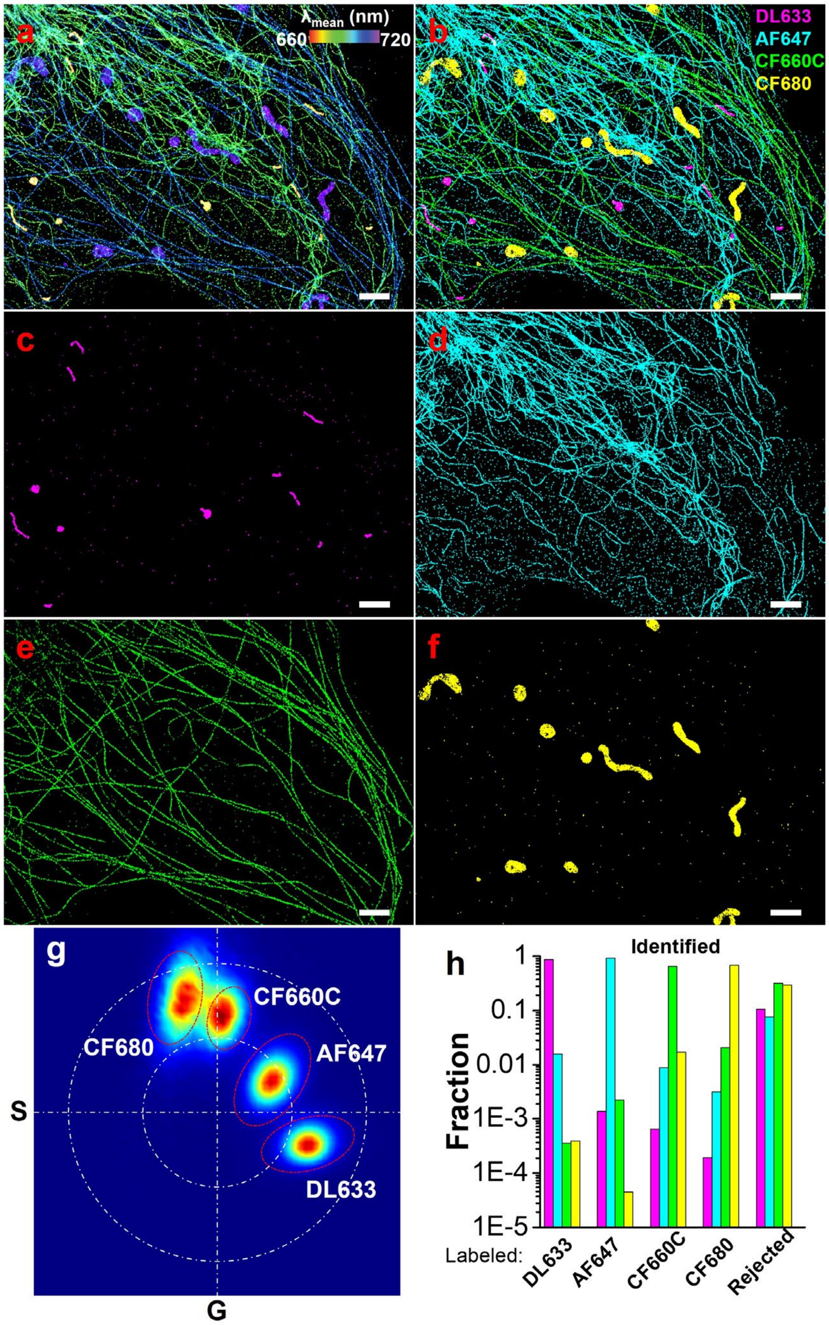

Figure S19. 4-color SP-STORM. (a) Hyperspectral dSTORM image of four proteins (i.e., PMP70, vimentin, α-tubulin, TOM20) labelled by DL633, AF647, CF660C, and CF680 in fixed COS-7 cells. The color denotes the spectral mean of single molecules. (b-f) 4-color dSTORM images of four proteins after classification of single molecules. (g) Boundary conditions in phasor plot were established from single-color labelled sample and used to separate the different dye molecules. The red dash dot line denotes 1.5-3.0 s.d. of distributions for each dye. The white dash dot line is for illustration location of dyes in the phasor plot with large and small circles as unit and half-unit circle respectively. (h) Color crosstalk between channels. Scale bar: 2 μm.

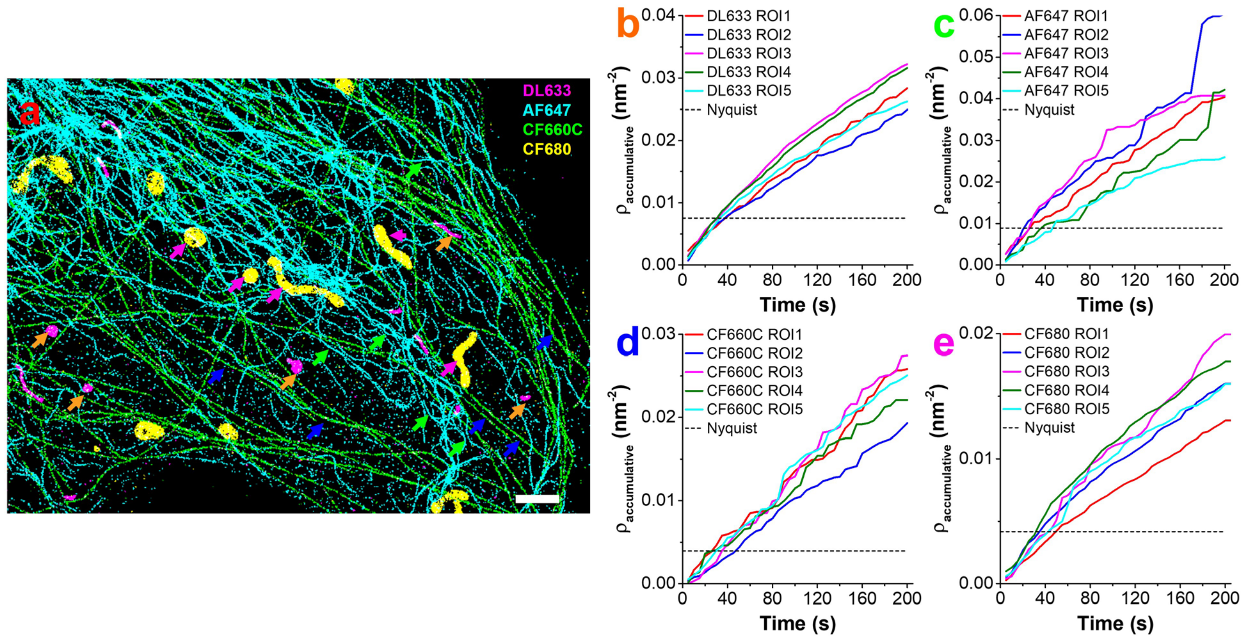

Figure S20. Density of single molecule locations versus imaging acquisition time for 4-color SP-STORM. (a) dSTORM image of four proteins (i.e., PMP70, vimentin, α-tubulin, TOM20) labelled by DL633, AF647, CF660C, and CF680 in fixed COS-7 cells. (b-e) Density plot of single molecule locations versus time for each dye as highlighted as colored arrows in (a). The short dash line represents minimal Nyquist densities[5] calculated from localization precisions, $\rho = (\frac{2}{2.355\sigma})^2$. Scale bar: 2 μm.

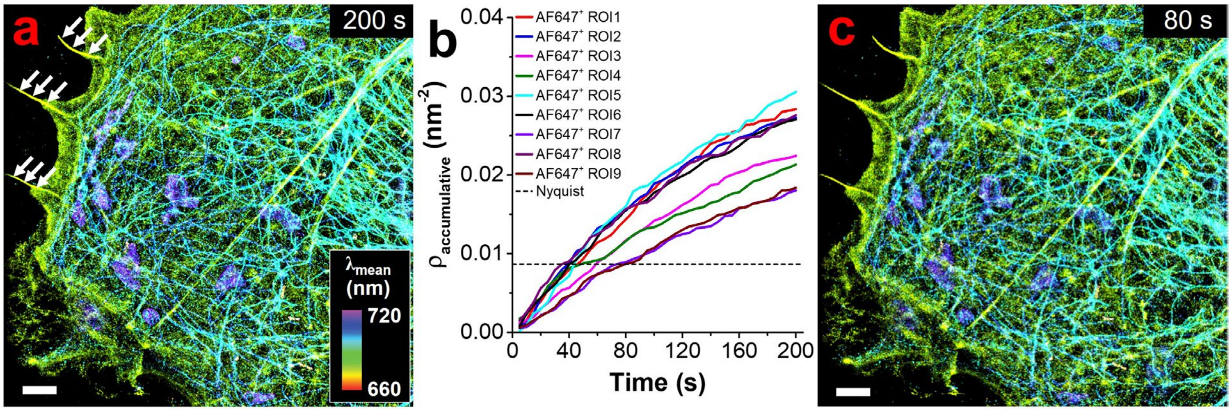

Figure S21. Density of single molecule locations versus imaging acquisition time for 5-color SP-STORM. (a) Hyperspectral dSTORM image of five proteins (i.e., PMP70, F-actin, vimentin, α-tubulin, TOM20) labelled by DL633, AF647$^+$, DL650, CF660C, and CF680 in fixed COS-7 cells. The color denotes the spectral mean of single molecules. (b) Density plot of single AF647$^+$ molecule locations versus time as highlighted as white arrows in (a). The short dash line represents minimal Nyquist densities calculated from localization precisions, $\rho = (\frac{2}{2.355\sigma})^2$. (c) Hyperspectral dSTORM image for a time segment of 80 seconds achieving minimal density based on the Nyquist criterion for AF647$^+$. The subcellular structures of labelled five proteins are already clearly distinguished. Scale bar: 2 μm.

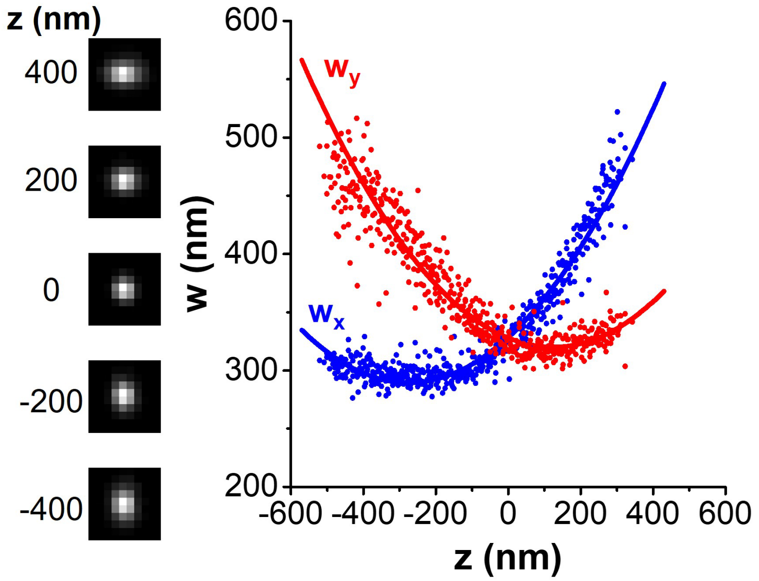

Figure S22. Calibrate the imaging system for 3D localization. Left: example images show the distinct PSF shapes of the same single emitter (40nm fluorescent beads) at different z positions. Right: the calibration curve of the image widths w_x and w_y as a function of z. The data was obtained from 12 fuorescent beads. The data were fit to a polynominal function (solid curve). The calibration curve was obtained using ThunderStorm ImageJ plugin.

Introducing the cylindrical lens close to the intermediate image plane induces an elliptical shape of point of spread function (PSF), where the width in x, y is z-position dependent. The relationship between PSF widths and z-position of single emitter were determined and used as the calibration curve for 3D localization of single dye molecules. Introducing cylindrical lens in the optical path changes the PSF in all three channels the same way, which causes no change on single-molecule spectral analysis. Same as 2D SP-STORM, photon number in all three channels were combined to 3D localize the position of single molecules, highlighting the photon usage efficiency of SP-STORM.

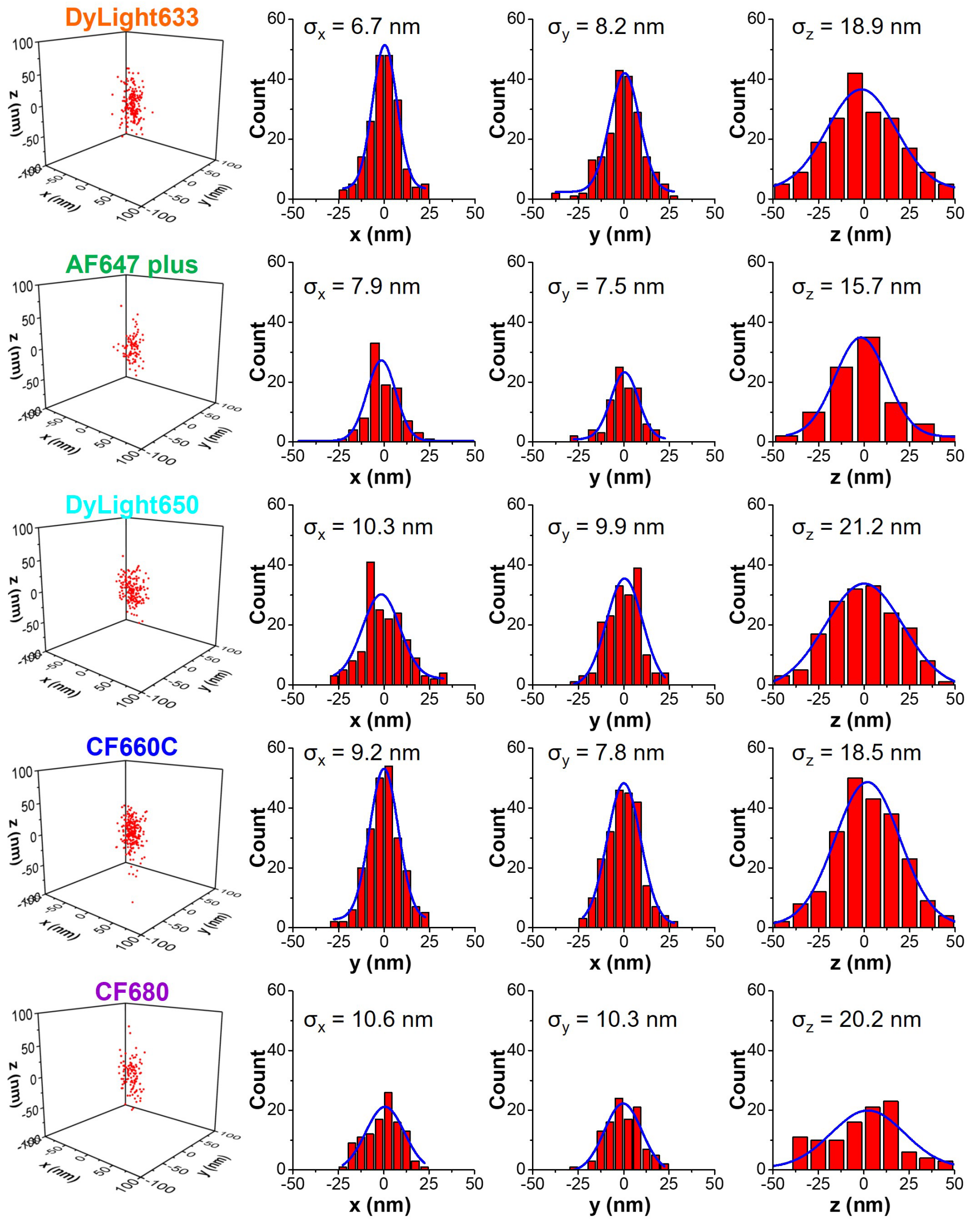

Figure S23. Evaluate 3D localization precision via small cluster analysis. Nonspecific bind dye labelled antibodies appear as smaller clusters were extracted from dSTORM images in each channel (Figure 5b-f). More than 20 smaller clusters with each cluster containing more than 10 localizations were obtained and aligned by their center mass for the cluster analysis. Histogram distributions in both x, y, and z dimensions of localizations in these clusters were then calculated. Fitting these histogram distributions with 1D Gaussian function gives the uncertainties of locations, which represents the localization precisions in the dSTORM images.

S9. Supporting Tables.

Table S1. The advantages and limitations of current multiplexed SMLM imaging techniques.

Method	Multiplexing capability	Spatial Resolution (nm)	Crosstalk	Throughput	Instrument complexity	# of Localizations	Major Limitations
Exchange PAINT[6]	Theoretically unlimited	Sub-5	Minimum	Slow can take days for whole cell multiplexing	Low (Standard TIRF microscope)	Not reported	Sequential imaging is inherently slow
Optimized PAINT[7]	Six	Sub-5	Minimum	30 minutes per structure			Limited number of orthogonal sequences
SUM-PAINT[8]	Theoretically unlimited	Sub-5	Minimum	30-plex in 30 hours			Sequential imaging is inherently slow
FLASH-PAINT[9]	Theoretically unlimited	Sub-5	Minimum	9-plex in 13 hours			Limited number of transient adaptors
Fluorogenic DNA-PAINT [10]	Two	Sub-5	Minimum	faster than standard DNA paint (1 hour for high localization precision.			Limited library of quencher-fluorophore pairs
Dual Objective + Dispersive Prism[11]	Four	Sub-10	< 2%	13 minutes	High (dual-objective setup: one channel for localization, one for spectrum)	$10^4 - 10^6$	Low molecular density per imaging frame to avoid spectral interference
Beam Splitter + Dispersive Prism[12]	Three	Sub-20	12%	20 minutes	Moderate (Need to add a prism in the imaging path)	$10^4 - 10^6$	Low photon efficiency; Low molecular density per imaging frame to avoid spectral interference
Gratings[13, 14]	Two	Sub-10	Not reported	20 minutes	Moderate (Need to add a grating in the imaging path)	$10^4 - 10^6$	Low photon efficiency; Low molecular density per imaging frame to avoid spectral interference
Ratiometric[15]	Four	Sub-20	20%	40 minutes	Moderate (Need to add dichroic mirrors in the imaging path)	$10^4 - 10^6$	Low photon efficiency, large spectral uncertainty for dyes with peaks at boundary conditions of spectral window
ExR-STORM[16]	Four	Sub-10	<3%	25 minutes	High (require multiple lasers and synchronization of switching excitation lasers and image projection by scanning mirror)	$10^4 - 10^6$	The sequential image registration limits the data acquisition speed
SP-STORM (This work)	Five	Sub-10	<5%	1 minute	Moderate (Imaging box with filters that have a sine and cosine transmission filter	$10^4 - 10^6$	not unlimited multiplexing

S10. References

(1) Batey, J. E.; Kim, G. W.; Yang, M.; Heffer, D. C.; Pott, E. D.; Giang, H.; Dong, B. High throughput spectrally resolved super-resolution fluorescence microscopy with improved photon usage. *Analyst* **2024**, *149* (10), 2801-2805.
(2) Ovesný, M.; Křížek, P.; Borkovec, J.; Švindrych, Z.; Hagen, G. M. ThunderSTORM: a comprehensive ImageJ plug-in for PALM and STORM data analysis and super-resolution imaging. *Bioinformatics* **2014**, *30* (16), 2389-2390.
(3) Batey, J. E.; Yang, M.; Giang, H.; Dong, B. Ultrahigh-Throughput Single-Particle Hyperspectral Imaging of Gold Nanoparticles. *Anal. Chem.* **2023**, *95* (13), 5479-5483.
(4) Dempsey, G. T.; Vaughan, J. C.; Chen, K. H.; Bates, M.; Zhuang, X. Evaluation of fluorophores for optimal performance in localization-based super-resolution imaging. *Nat. Methods* **2011**, *8* (12), 1027-1036.
(5) Shroff, H.; Galbraith, C. G.; Galbraith, J. A.; Betzig, E. Live-cell photoactivated localization microscopy of nanoscale adhesion dynamics. *Nat. Methods* **2008**, *5* (5), 417-423.
(6) Jungmann, R.; Avendaño, M. S.; Woehrstein, J. B.; Dai, M.; Shih, W. M.; Yin, P. Multiplexed 3D cellular super-resolution imaging with DNA-PAINT and Exchange-PAINT. *Nat. Methods* **2014**, *11* (3), 313-318.
(7) Strauss, S.; Jungmann, R. Up to 100-fold speed-up and multiplexing in optimized DNA-PAINT. *Nat. Methods* **2020**, *17* (8), 789-791.
(8) Unterauer, E. M.; Shetab Boushehri, S.; Jevdokimenko, K.; Masullo, L. A.; Ganji, M.; Sograte-Idrissi, S.; Kowalewski, R.; Strauss, S.; Reinhardt, S. C. M.; Perovic, A.; et al. Spatial proteomics in neurons at single-protein resolution. *Cell* **2024**, *187* (7), 1785-1800.e1716.
(9) Schueder, F.; Rivera-Molina, F.; Su, M.; Marin, Z.; Kidd, P.; Rothman, J. E.; Toomre, D.; Bewersdorf, J. Unraveling cellular complexity with transient adapters in highly multiplexed super-resolution imaging. *Cell* **2024**, *187* (7), 1769-1784.e1718.
(10) Chung, K. K. H.; Zhang, Z.; Kidd, P.; Zhang, Y.; Williams, N. D.; Rollins, B.; Yang, Y.; Lin, C.; Baddeley, D.; Bewersdorf, J. Fluorogenic DNA-PAINT for faster, low-background super-resolution imaging. *Nat. Methods* **2022**, *19* (5), 554-559.
(11) Zhang, Z.; Kenny, S. J.; Hauser, M.; Li, W.; Xu, K. Ultrahigh-throughput single-molecule spectroscopy and spectrally resolved super-resolution microscopy. *Nat. Methods* **2015**, *12*, 935.
(12) Mlodzianoski, M. J.; Curthoys, N. M.; Gunewardene, M. S.; Carter, S.; Hess, S. T. Super-Resolution Imaging of Molecular Emission Spectra and Single Molecule Spectral Fluctuations. *PloS one* **2016**, *11* (3), e0147506-e0147506.
(13) Martens, K. J. A.; Gobes, M.; Archontakis, E.; Brillas, R. R.; Zijlstra, N.; Albertazzi, L.; Hohlbein, J. Enabling Spectrally Resolved Single-Molecule Localization Microscopy at High Emitter Densities. *Nano Lett.* **2022**, *22* (21), 8618-8625.
(14) Dong, B.; Almassalha, L.; Urban, B. E.; Nguyen, T.-Q.; Khuon, S.; Chew, T.-L.; Backman, V.; Sun, C.; Zhang, H. F. Super-resolution spectroscopic microscopy via photon localization. *Nat. Commun.* **2016**, *7* (1), 12290.
(15) Testa, I.; Wurm, C. A.; Medda, R.; Rothermel, E.; von Middendorf, C.; Fölling, J.; Jakobs, S.; Schönle, A.; Hell, S. W.; Eggeling, C. Multicolor Fluorescence Nanoscopy in Fixed and Living Cells by Exciting Conventional Fluorophores with a Single Wavelength. *Biophys. J.* **2010**, *99* (8), 2686-2694.
(16) Wu, W.; Luo, S.; Fan, C.; Yang, T.; Zhang, S.; Meng, W.; Xu, T.; Ji, W.; Gu, L. Tetra-color superresolution microscopy based on excitation spectral demixing. *Light Sci. Appl.* **2023**, *12* (1), 9.
